# Supplementary figures and images for: Dissecting the shared genetic architecture of bipolar disorder, major depressive disorder, and attention-deficit hyperactivity disorder
Source: PLoS One. 2026 Feb 23;21(2):e0333571. doi: 10.1371/journal.pone.0333571 (PMC12928397; doi:10.1371/journal.pone.0333571)

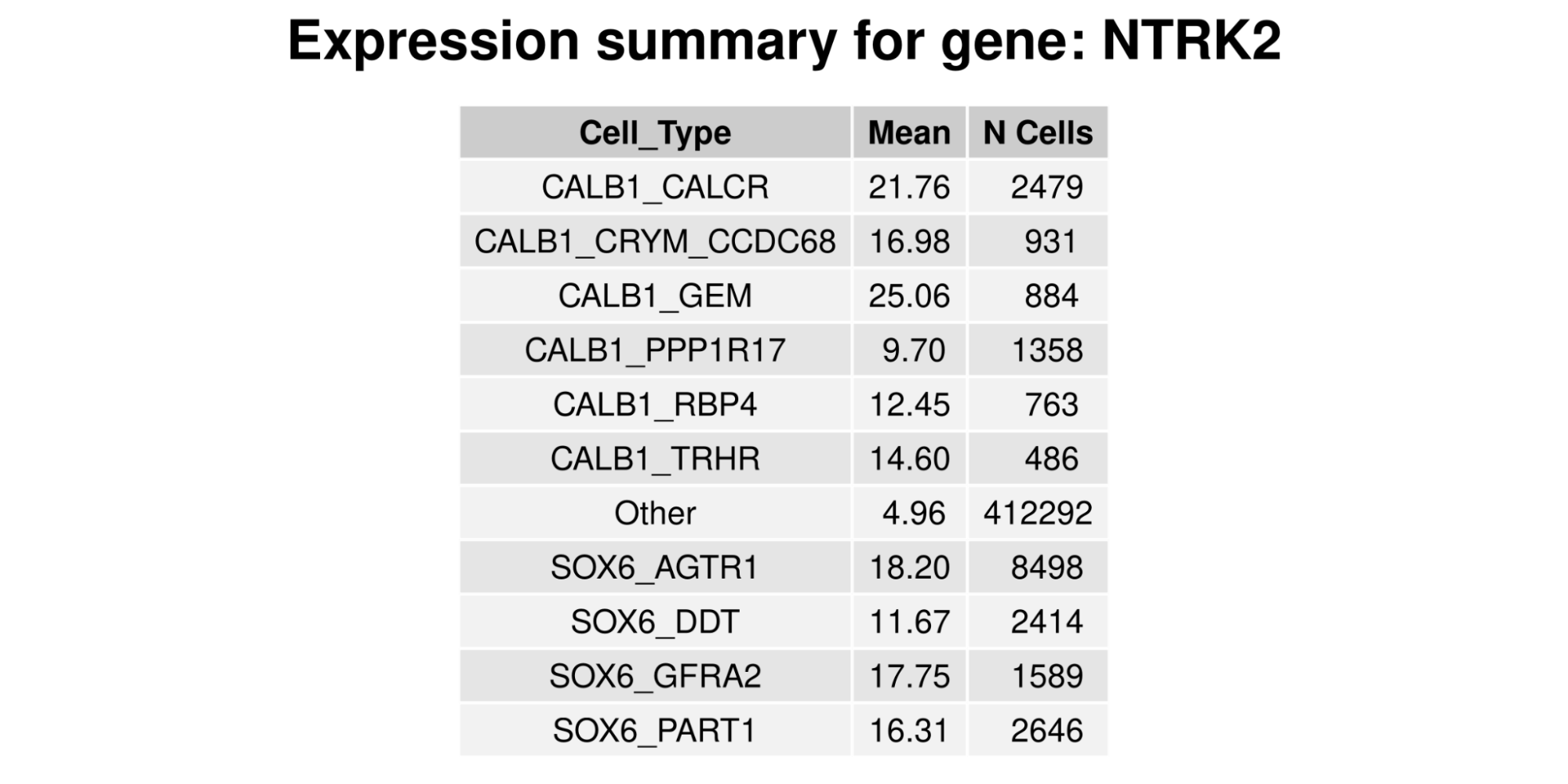

Supplement: S1 Fig — (TIFF) [file pone.0333571.s001.tiff]

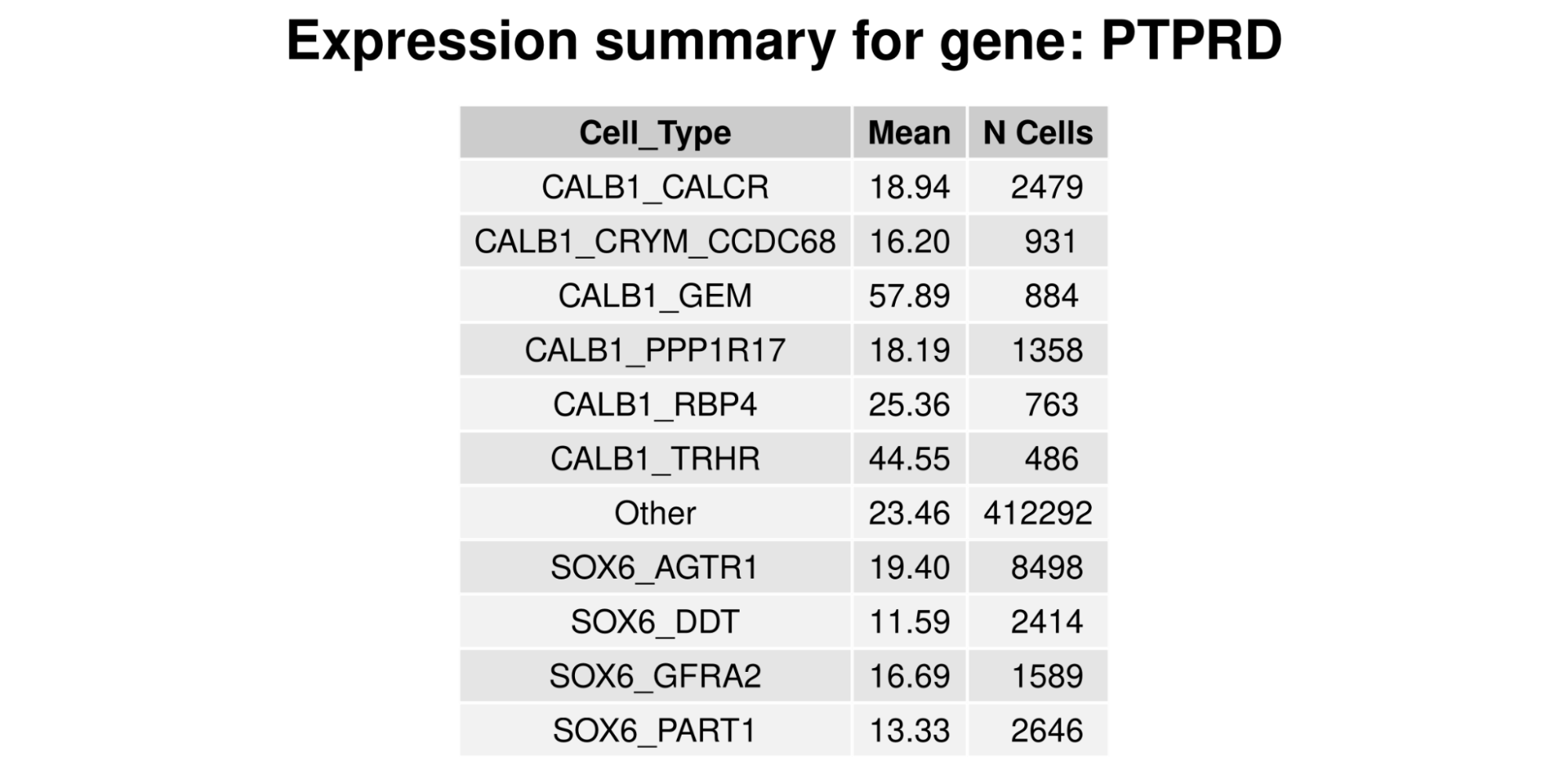

Supplement: S2 Fig — (TIFF) [file pone.0333571.s002.tiff]

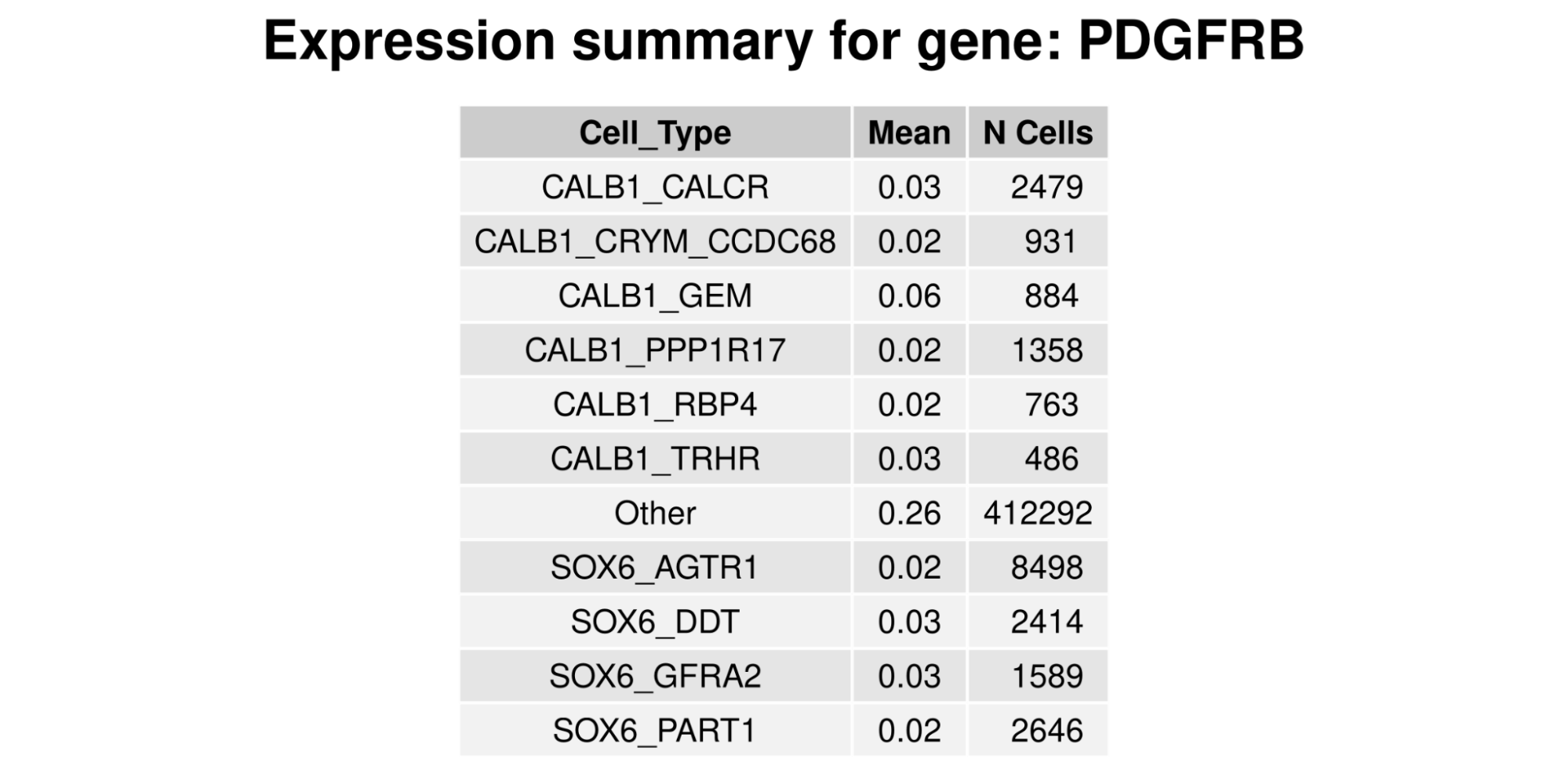

Supplement: S3 Fig — (TIFF) [file pone.0333571.s003.tiff]

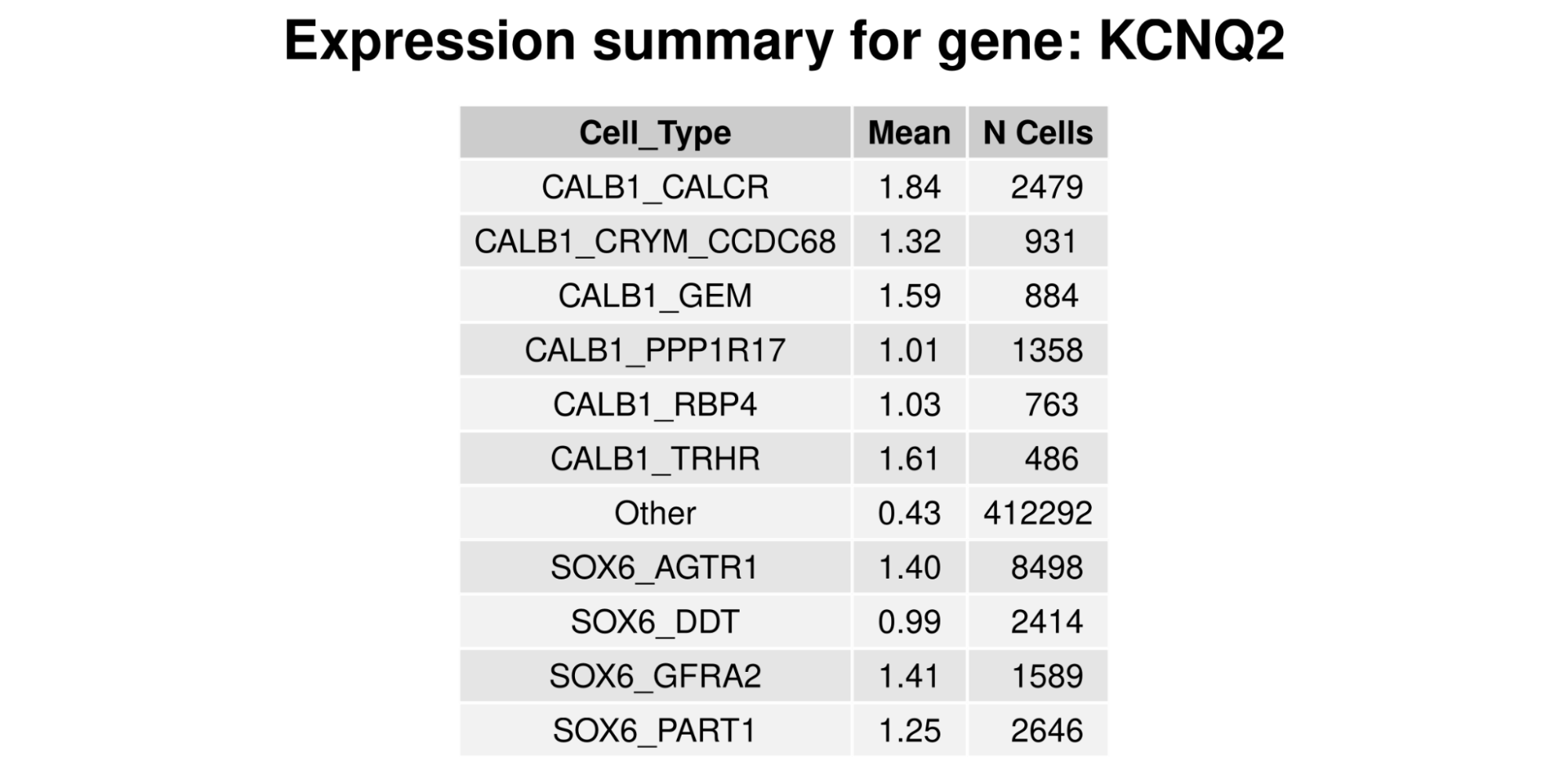

Supplement: S4 Fig — (TIFF) [file pone.0333571.s004.tiff]

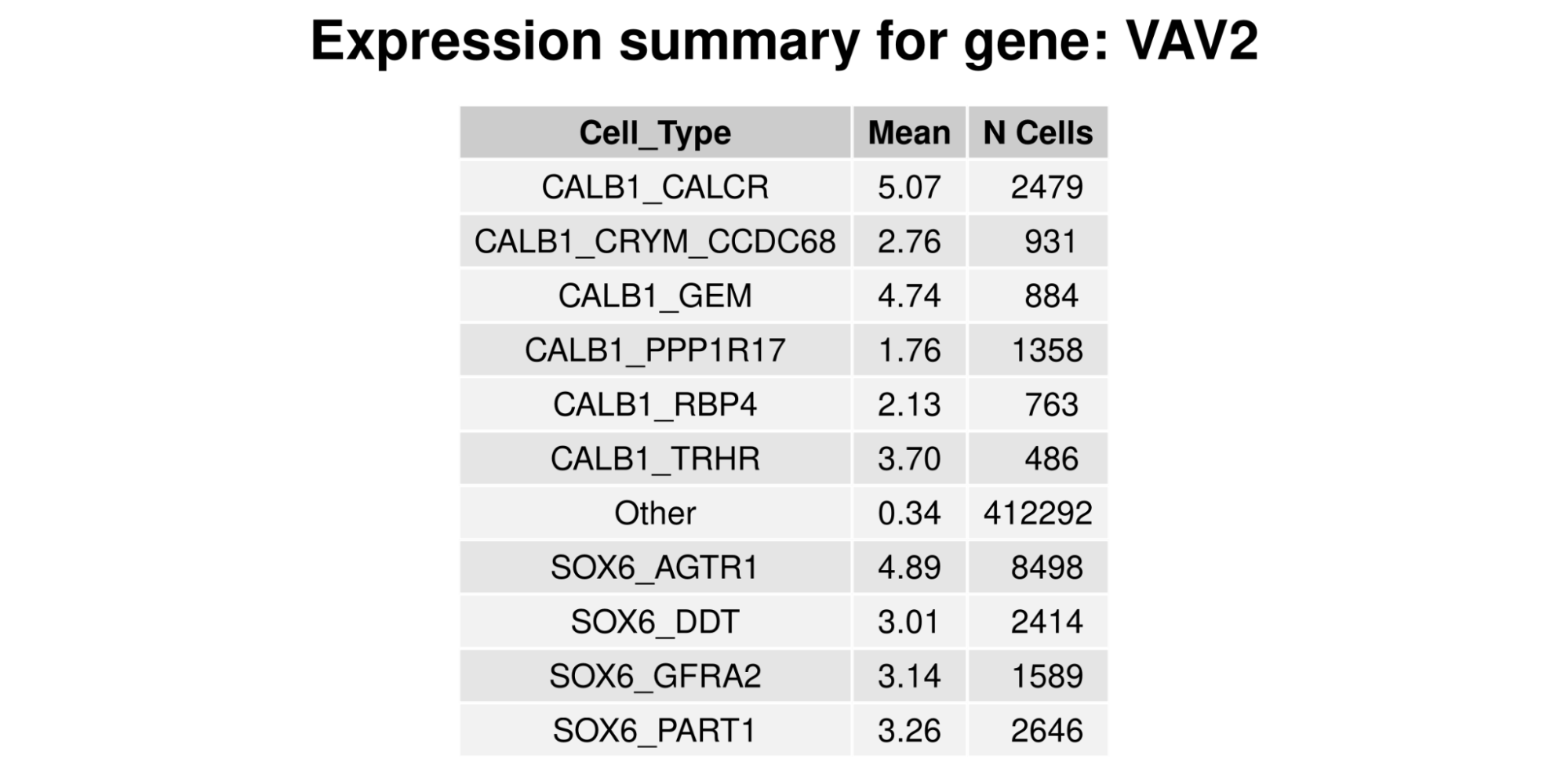

Supplement: S5 Fig — (TIFF) [file pone.0333571.s005.tiff]

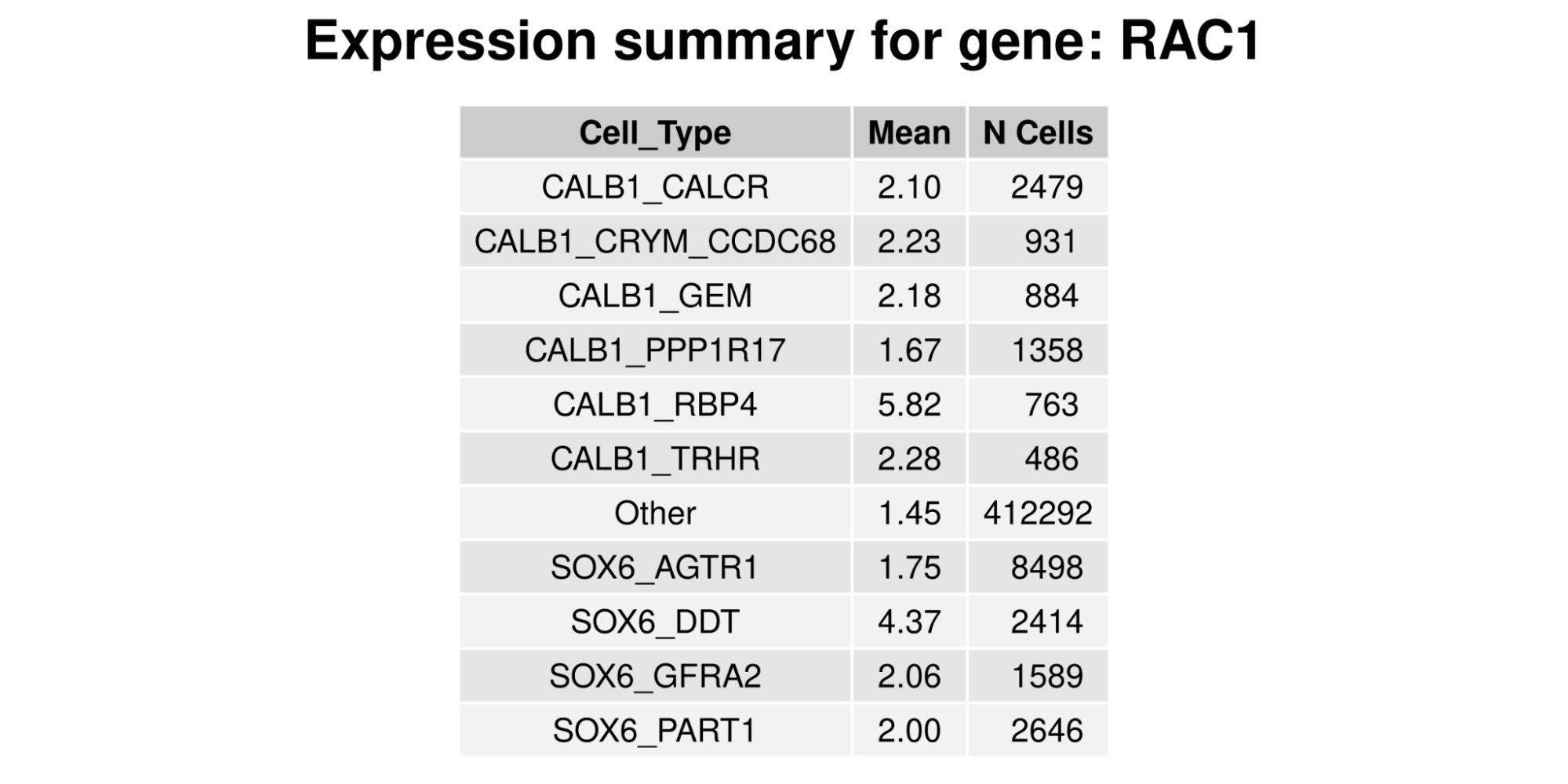

Supplement: S6 Fig — (TIFF) [file pone.0333571.s006.tiff]

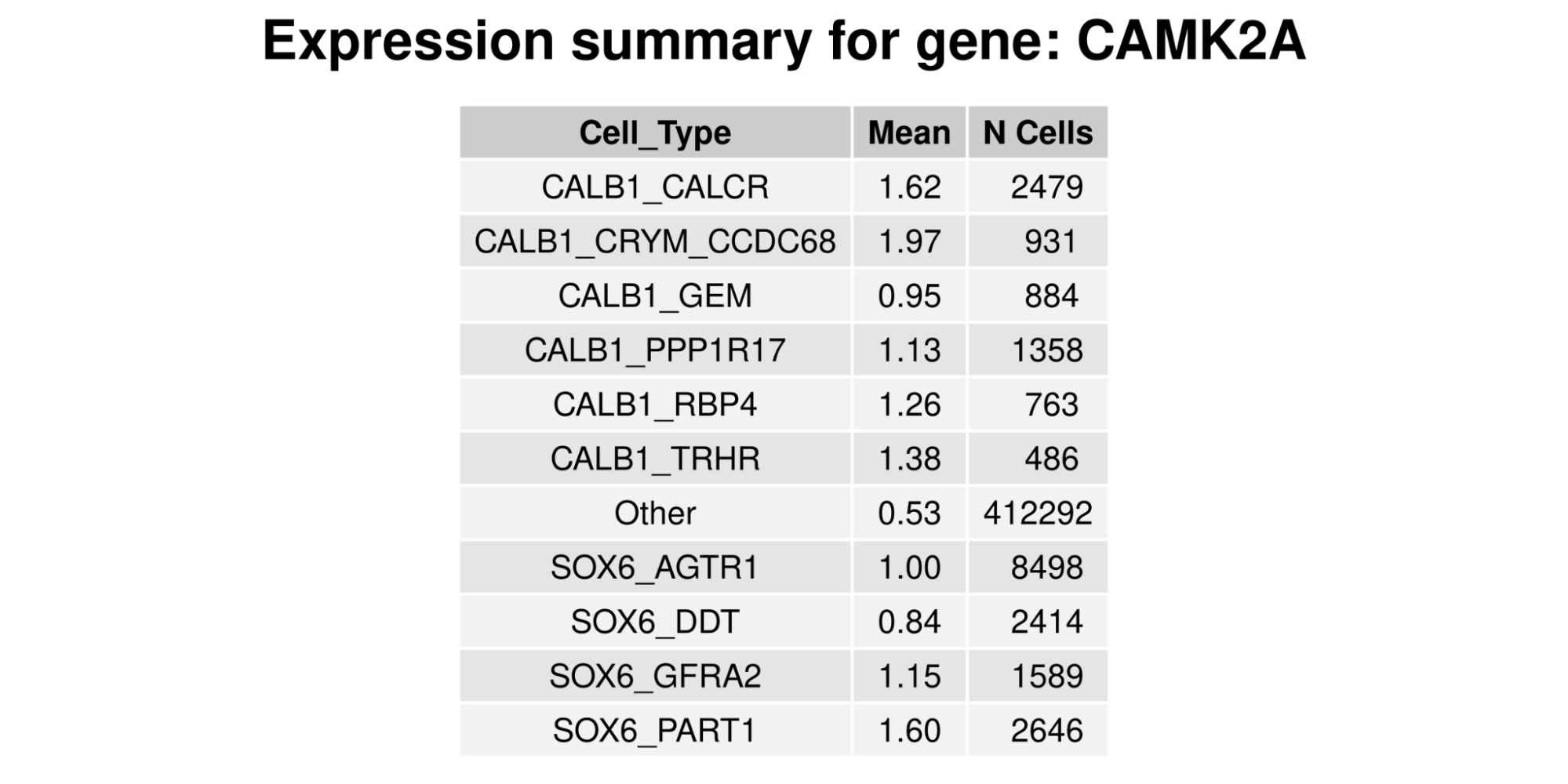

Supplement: S7 Fig — (TIFF) [file pone.0333571.s007.tiff]

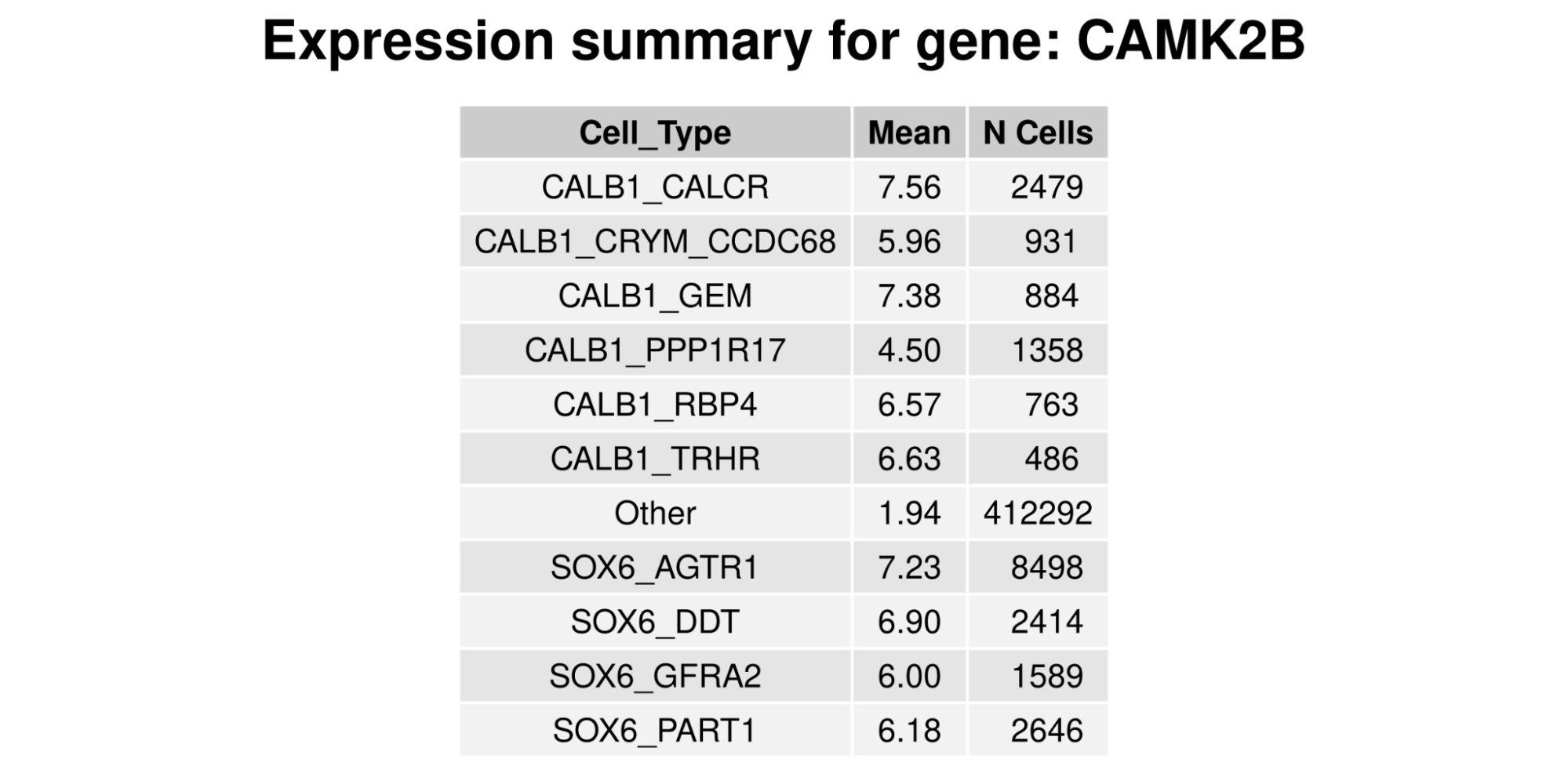

Supplement: S8 Fig — (TIFF) [file pone.0333571.s008.tiff]

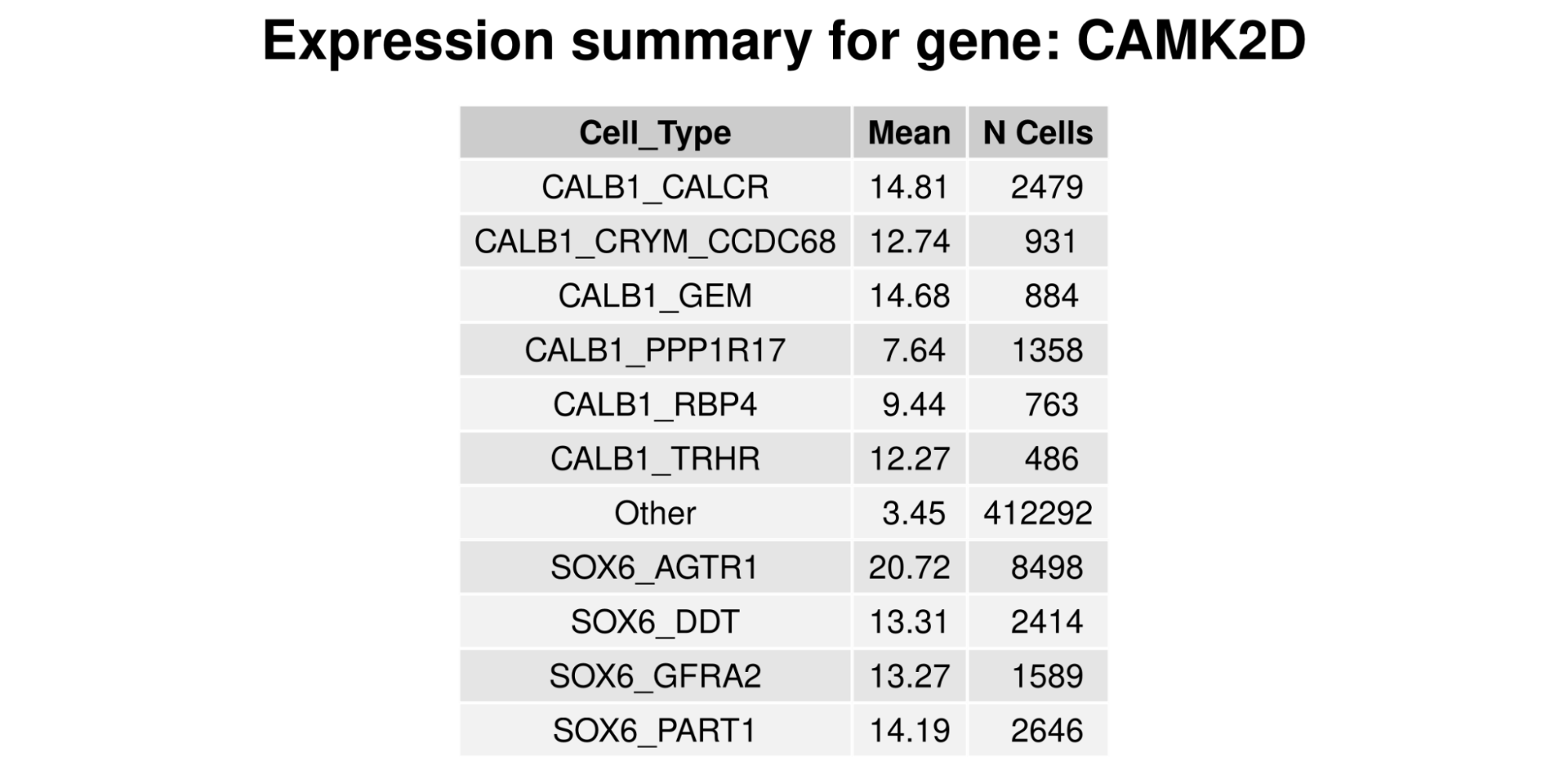

Supplement: S9 Fig — (TIFF) [file pone.0333571.s009.tiff]

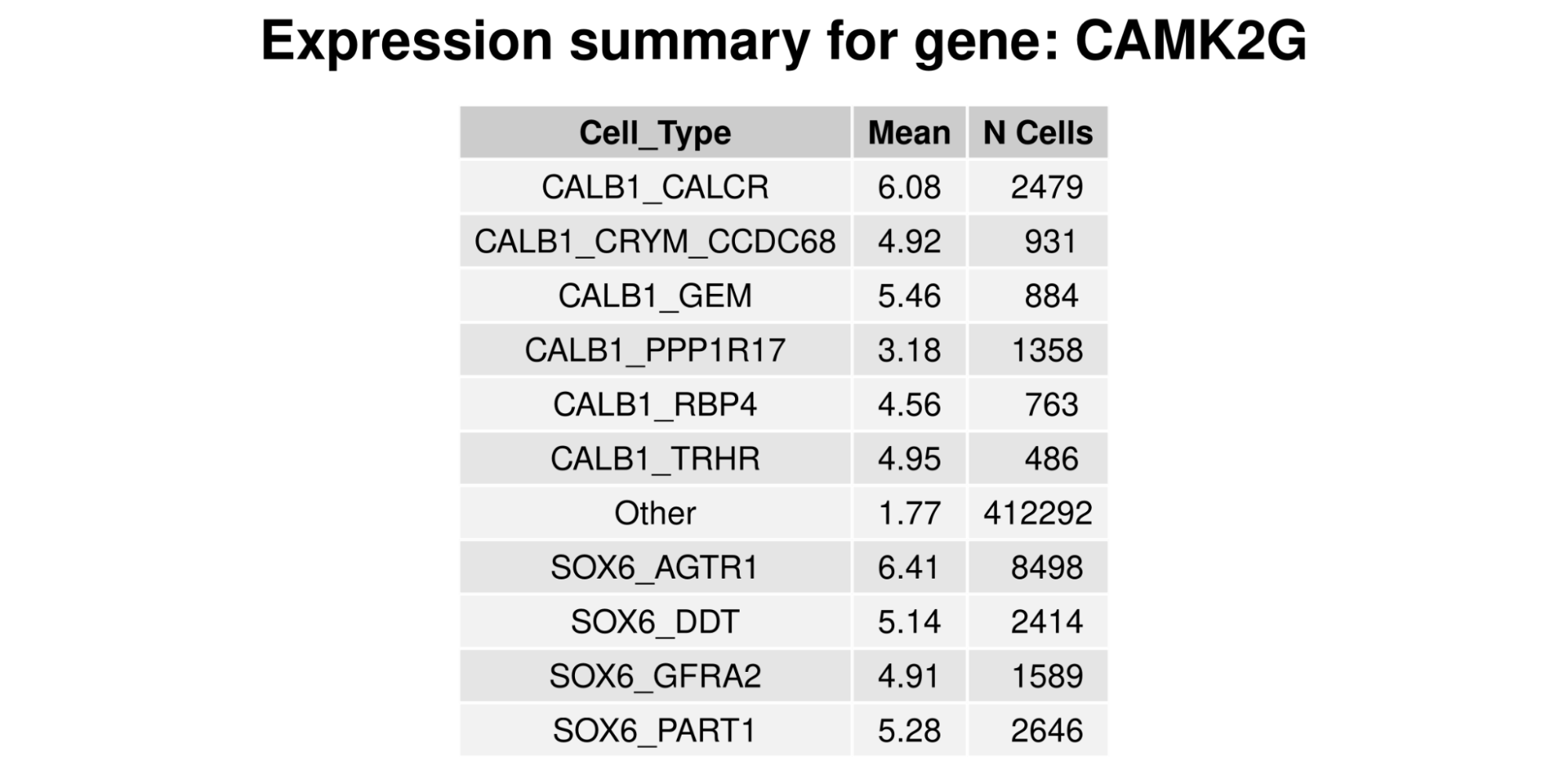

Supplement: S10 Fig — (TIFF) [file pone.0333571.s010.tiff]

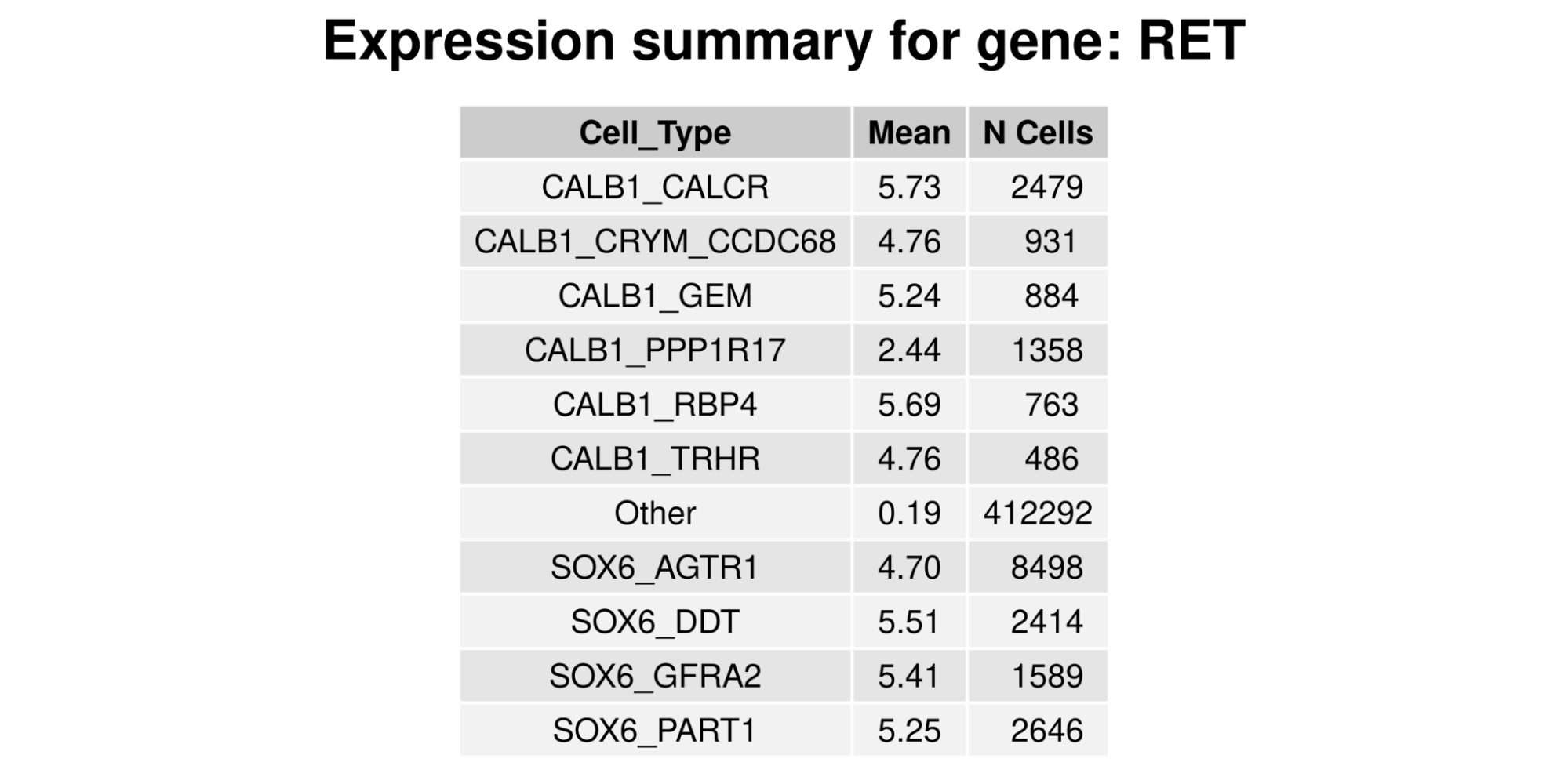

Supplement: S11 Fig — (TIFF) [file pone.0333571.s011.tiff]

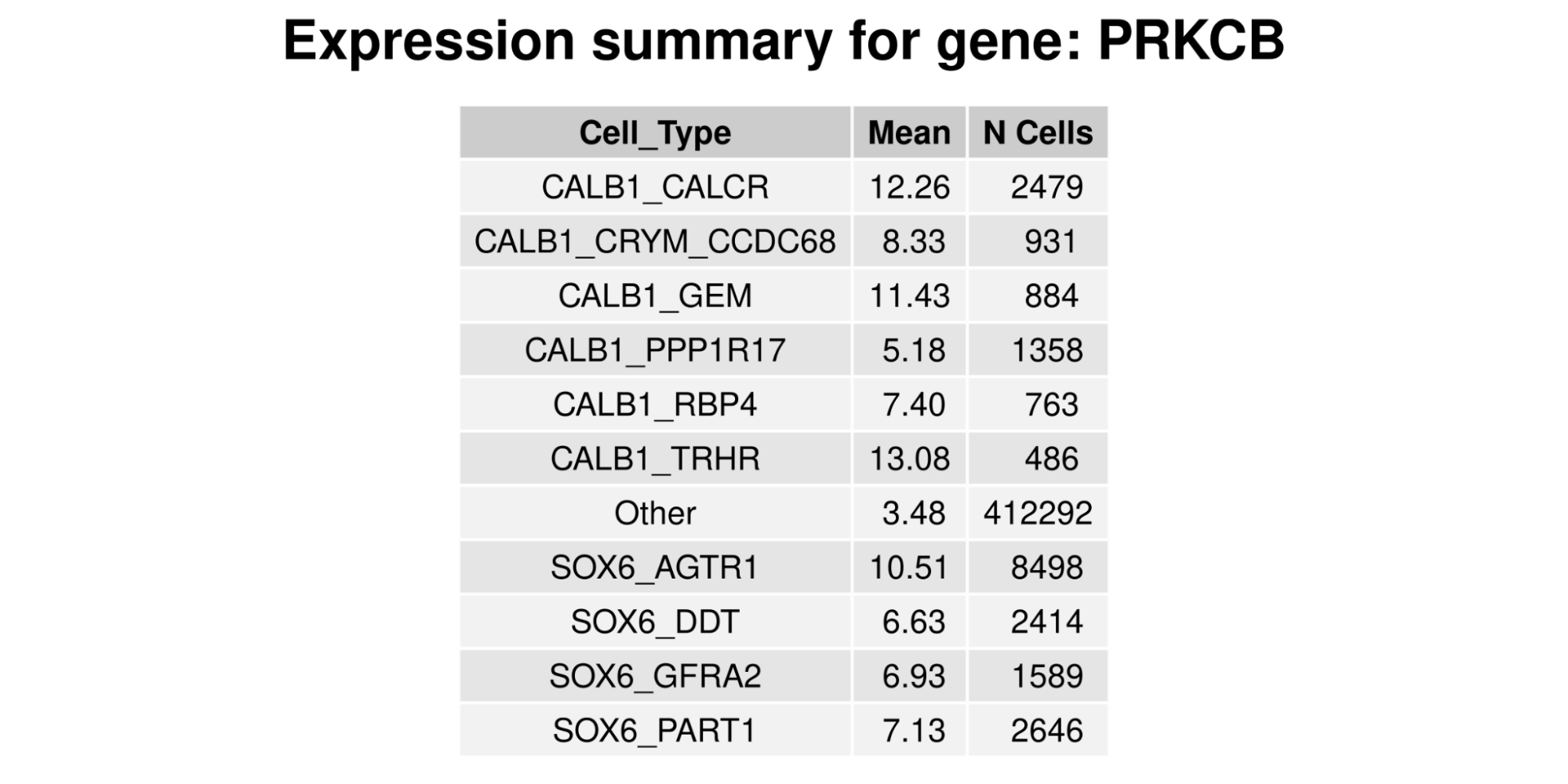

Supplement: S12 Fig — (TIFF) [file pone.0333571.s012.tiff]

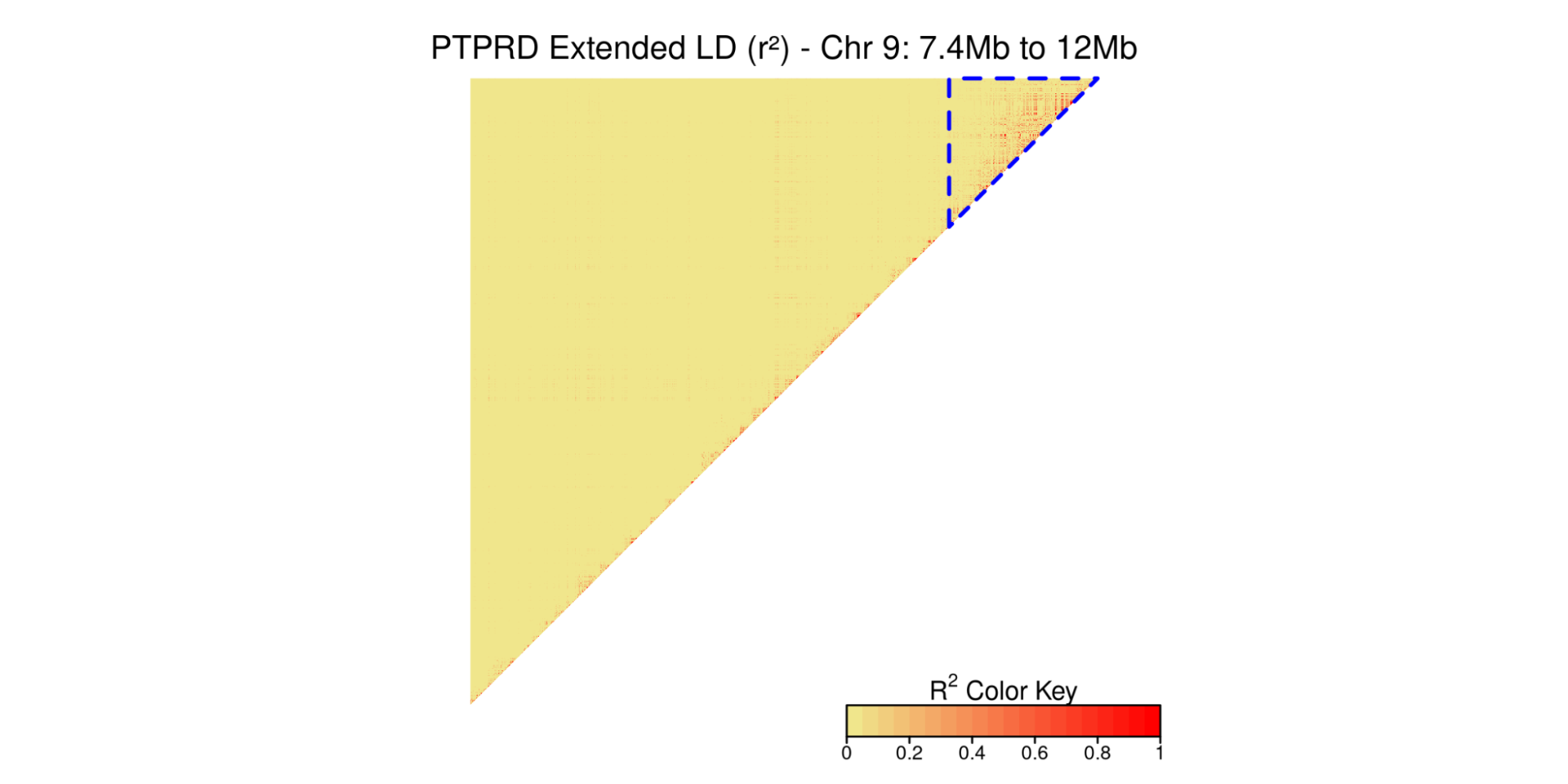

Supplement: S13 Fig — (TIFF) [file pone.0333571.s013.tiff]

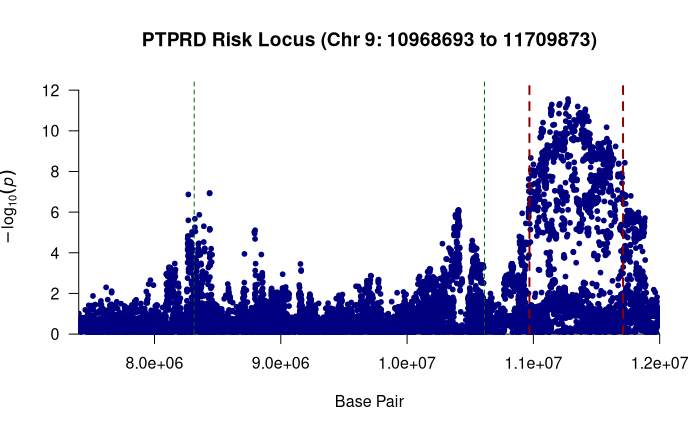

Supplement: S14 Fig — (TIFF) [file pone.0333571.s014.tiff]

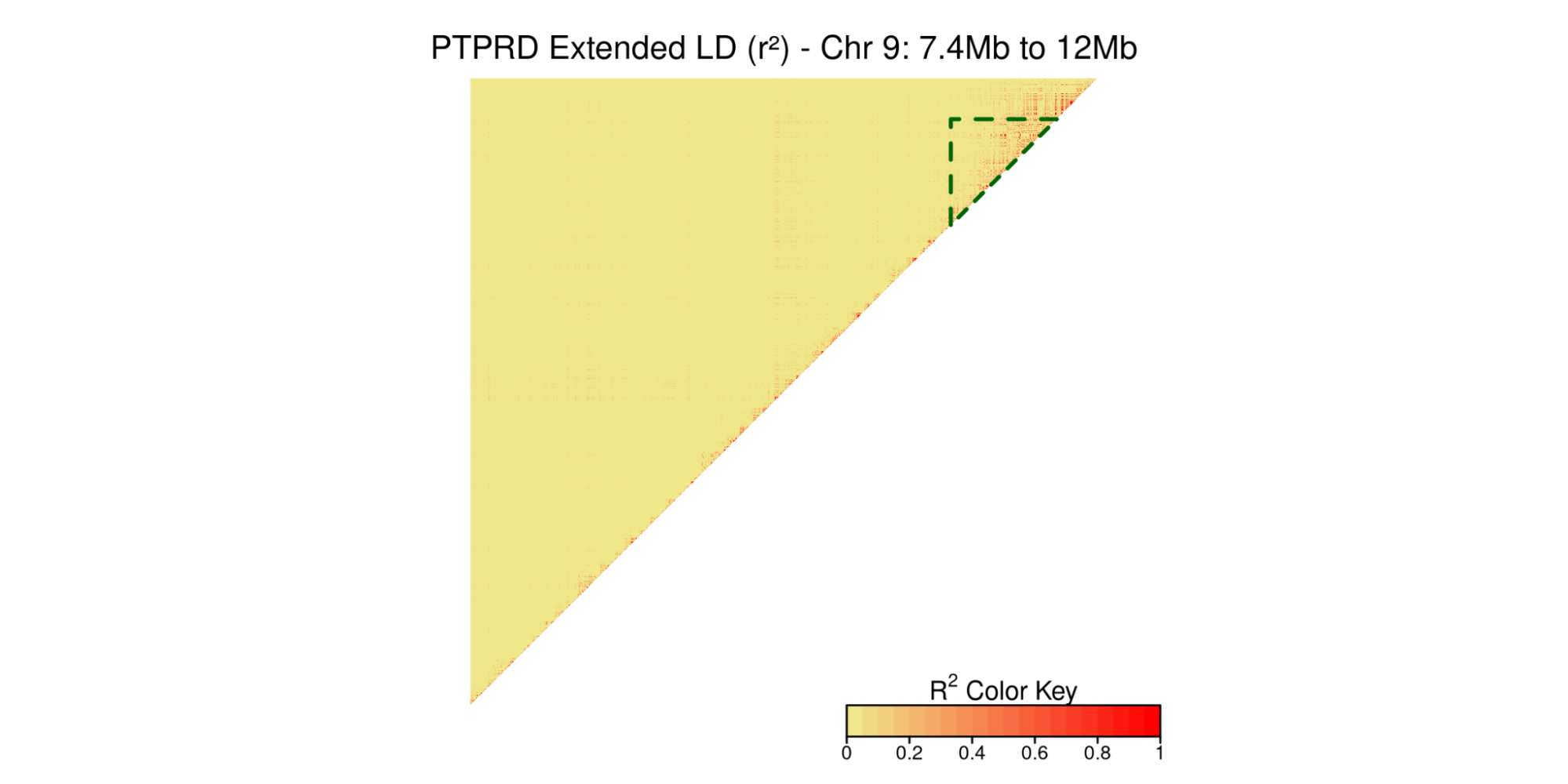

Supplement: S15 Fig — (TIFF) [file pone.0333571.s015.tiff]

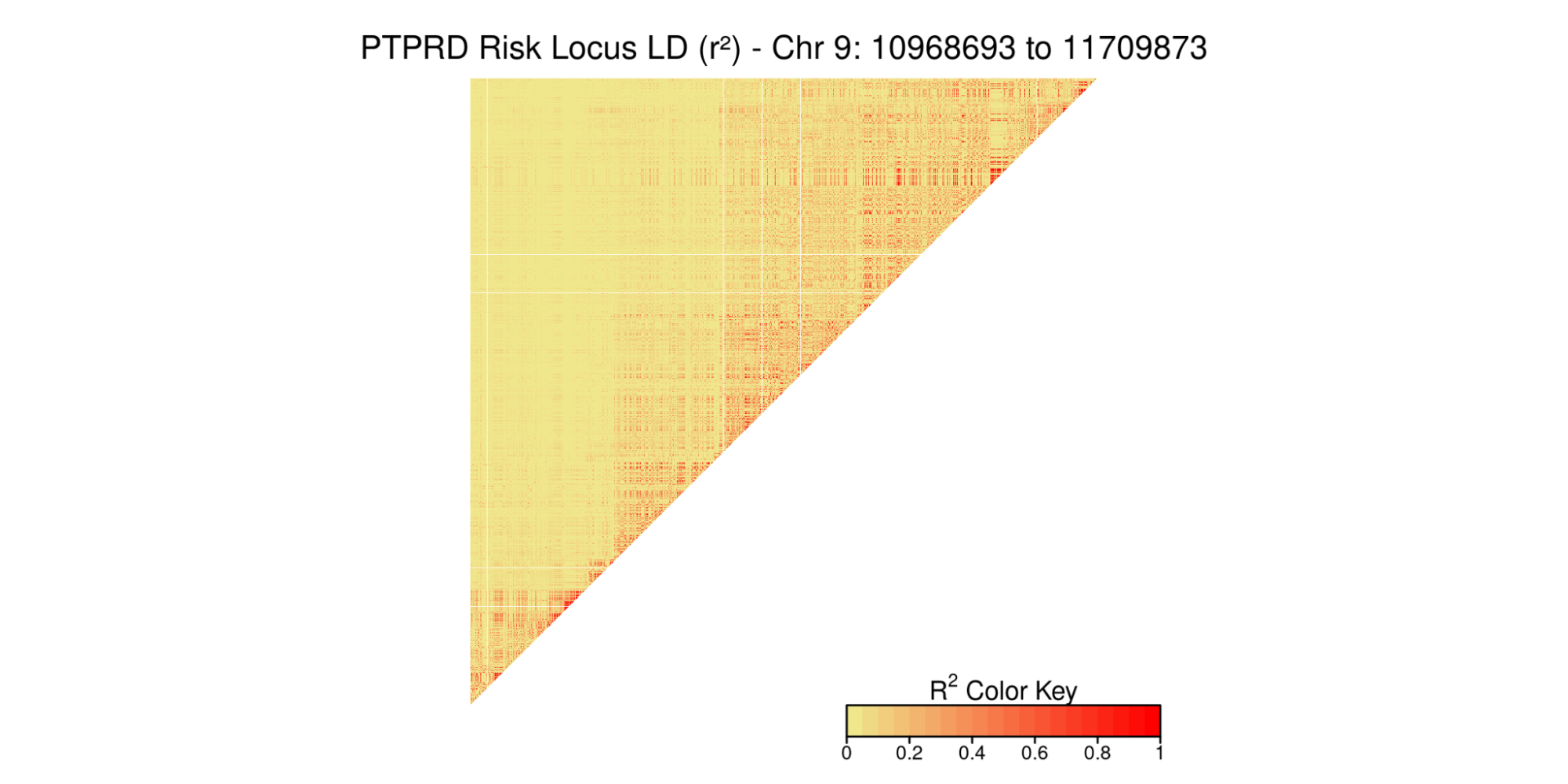

Supplement: S16 Fig — (TIFF) [file pone.0333571.s016.tiff]
