# Supplementary material for: Dissecting the shared genetic architecture of bipolar disorder, major depressive disorder, and attention-deficit hyperactivity disorder
Source: PLoS One. 2026 Feb 23;21(2):e0333571. doi: 10.1371/journal.pone.0333571 (PMC12928397; doi:10.1371/journal.pone.0333571)
Supplement: S1 Appendix — (DOCX) [file pone.0333571.s051.docx]

**Supplementary Note 1.**

Univariate and or multivariate GWAS summary statistics are not sufficient to reveal the mechanisms that underlie pathogenesis. Rather, it is the downstream analysis of these genetic datasets that reveal how complex traits may develop at the biochemical level. Using summary statistics as input, a majority of gene-based tests assign variants based on reference genome gene position, often including SNPs within pre-specified upstream and downstream windows to capture potential regulatory elements. However, such methodologies are not capable of evaluating cell type-specific gene associations. Nuclear organization is highly cell type-dependent and is extremely correlated to the differences in transcription across cell types [1]. There is significant variation in nuclear volume, chromatin scaling, chromosome territory size, overall 3D chromatin structures of inactive and active chromatin, radial positioning and consequent differences in transcriptional activity across cell types, even across neuronal subclasses [2]. There is variation amongst the group(s) of SNPs that affect any given gene’s expression across cell types. Due to this immense limitation, there is a lack of cell type-specific gene-based interpretation of GWAS. To date, H-MAGMA is the only gene-analysis framework that helps overcome cell type-dependent nuclear organization limitations by leveraging chromatin interactions to map noncoding variants to genes. However, open-sourced Hi-C datasets are extremely sparse, attenuating our ability to view genetic risk through a “cell-specific” lens. Nonetheless, by mapping risk genes in neurotransmitter-specific neurons from the genetic data of psychiatric disorders driven by aberrant neurotransmission, one inherently targets genes that are core to the cell type’s signaling machinery [3]. Quantifying risk genes for specific cell types will add specificity and depth to research, likely expediting the unraveling of complex trait pathogenesis.

**Supplementary Note 2. Disregarding the cell adhesion aspect of *PTPRD***

We first investigated the role of *PTPRD*’s cell adhesion mediating fibronectin III and Ig domains, as the receptor is highly implicated in synaptic formation and differentiation (Cortés et al. 2024). However, combined knockout of 3 receptor type protein tyrosine phosphatases, *PTPRD*, *PTPRS*, and *PTPRF* yielded intact synapse structure and function in cultured hippocampal neurons [4]. In another study, *PTPRD* KO mice exhibited normal excitatory and inhibitory synaptic functions [5]. The literature suggests that *PTPRD* mediated cell adhesion contributes redundantly to the overall anatomy of the neuronal synapse, thus we decided not to pursue the topic further in our biological annotation.

**Supplementary Note 3. *PTPRD*/*TrkB* and *PDGFRB***

Substrate-trapping pulldowns with *PTPRD’s* D1 phosphatase domain have supported direct dephosphorylation interactions with the receptor protein kinases, platelet derived growth factor beta (*PDGFRB*) and tropomyosin receptor type beta (*TrkB* encoded by the *NTRK2* gene) in mice. These findings are consistent with recent literature, naming *PDGFRB* and *TrkB* as substrates of *PTPRD* [6]. In the absence of *PTPRD*, *TrkB* and *PDGFRB*, which potentiate downstream the *MEK*-*ERK* pathway, remain hyperactivated. Higher concentrations of downstream effectors, *MEK* and *ERK*, are observed in *PTPRD* KO/HET mice when compared to the WT (Toita et al. 2020). In light of these developments, we explored the intricacies of the two *PTPRD* substrates, *TrkB* and *PDGFRB*, and the potential of their downstream effectors to hold functional relevance in dopamine neurons.

**Supplementary Note 4. Disregarding *PDGFRB***

The single-cell, dopamine neuron RNA expression dataset curated by Kamath et al. 2022 [7] was used as a helping guide for the pathway analysis of *PTPRD*, allowing us to rule out any candidates that are expressed in negligible amounts in human dopamine neurons. Given the negligible expression of *PDGFRB* in dopamine neurons (Supplementary Fig 3), the gene was excluded from analysis as a downstream effector of *PTPRD* signaling. Supplementary Figs 1-2 display the mean RNA expression (Unique molecular identifiers) for *PTPRD* and *NTRK2* for 10 subclasses of clustered dopamine neurons. Both genes appear to be highly expressed across dopamine neuron subclasses, thus *NTRK2* was subjected to further analysis in the context of a downstream effector of *PTPRD*.


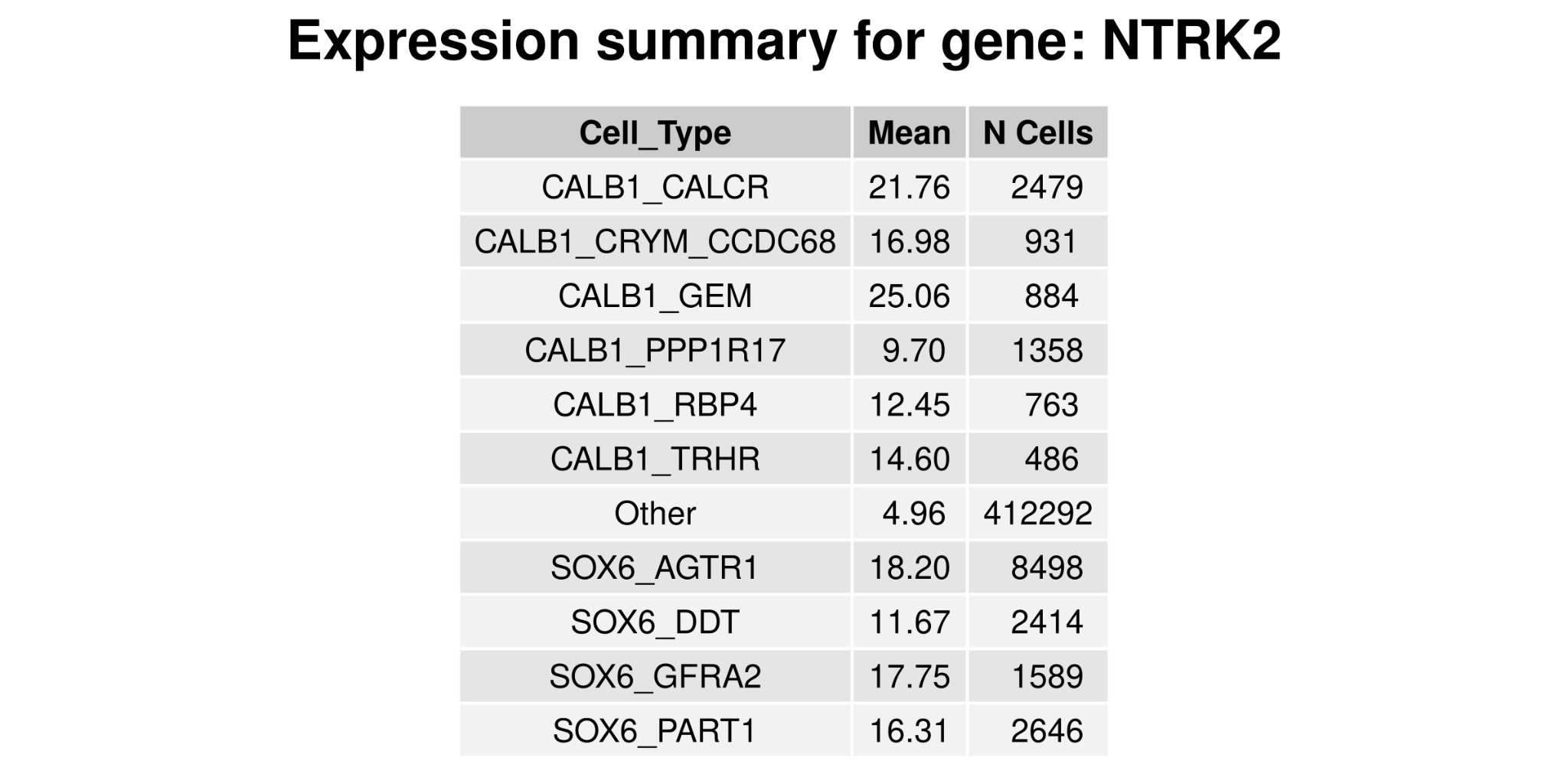
Supplementary Fig 1. RNA expression tables displaying mean UMIs for *NTRK2* in 10 subclasses of human dopamine neuron clusters from the Kamath et. al 2022 dataset.
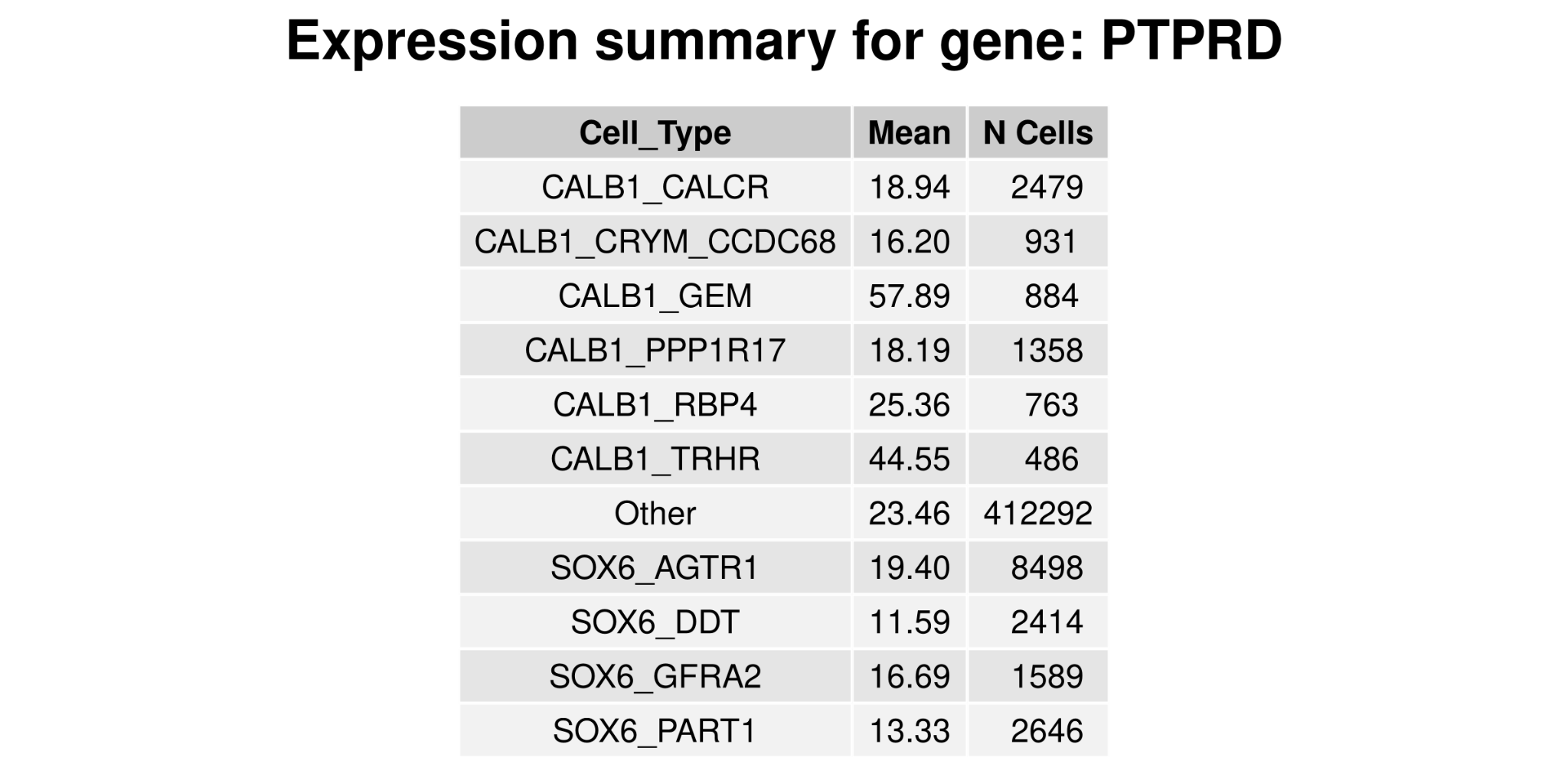


Supplementary Fig 2. RNA expression tables displaying mean UMIs for *PTPRD* in 10 subclasses of human dopamine neuron clusters from the Kamath et. al 2022 dataset.


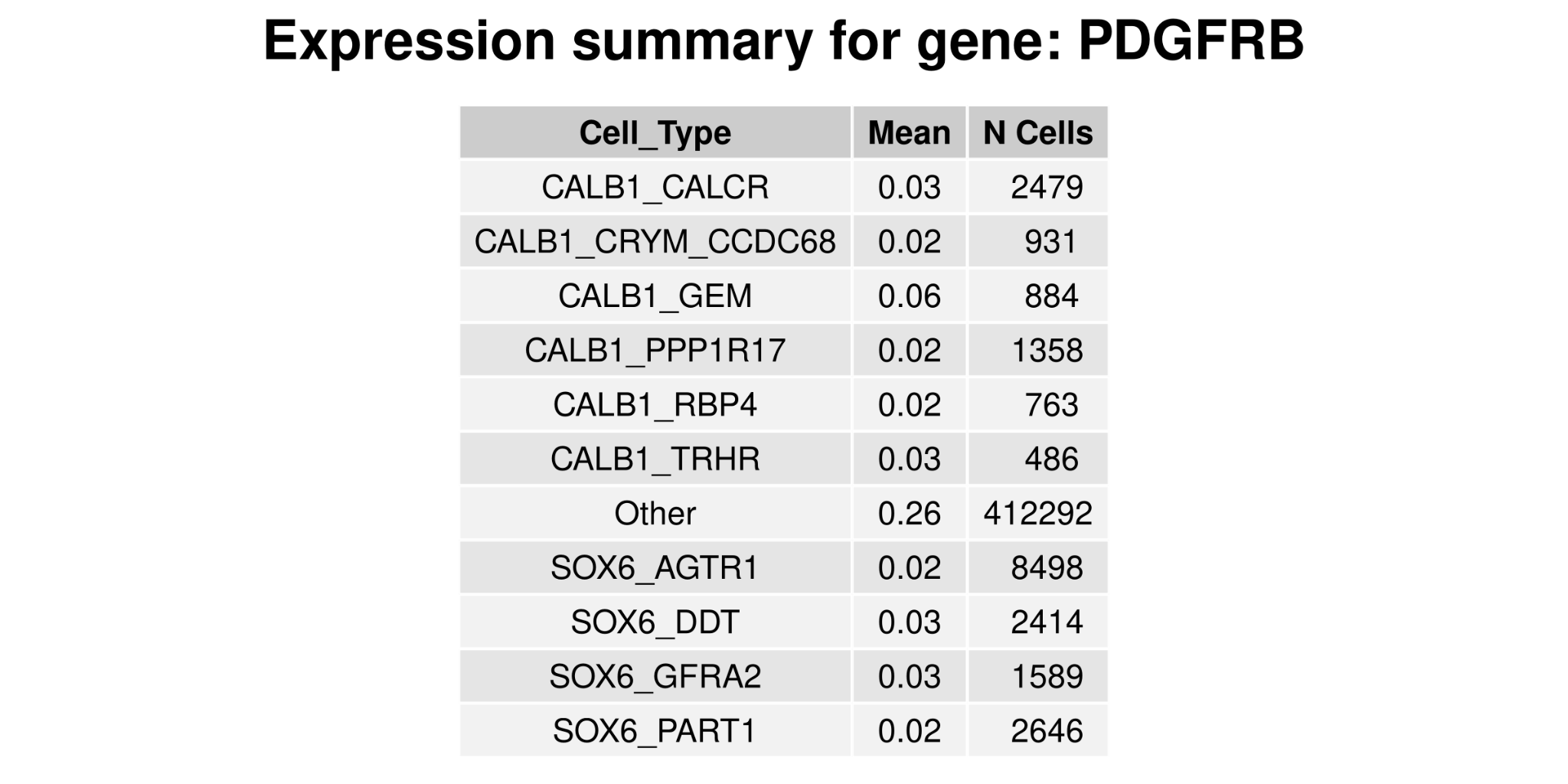


Supplementary Fig 3. RNA expression tables displaying mean UMIs for *PDGFRB* in 10 subclasses of human dopamine neuron clusters from the Kamath et. al 2022 dataset.

**Supplementary Note 5. *ERK1/2* signaling involved in regulation *KCNQ2* K+ ion channels**

Given the propensity of *PTPRD* inhibition to increase *MEK*/*ERK* signaling, we reviewed neuron associated *ERK1*/2 literature to gain insights into pathways that may hold functional relevance in DA neurons. *ERK1/2* is heavily implicated in the LTP of neurons in the nucleus accumbens in response to dopaminergic signaling [8]. Dopamine-mediated signaling of the D1 receptor activates the *PKA*/*RAP1*/*ERK* cascade in medium spiny neurons of the nucleus accumbens to regulate membrane excitability [8]. In HEK293 cells in vivo, *ERK* inhibits KCNQ-mediated K+ current via direct phosphorylation of *KCNQ2* ion channels at Ser414 and Ser476. Additionally, D1 receptor agonist induced inhibition of the KCNQ-mediated currents were attenuated by *ERK* inhibition and conditional deletion of *KCNQ2* in medium spiny neurons (Tsuboi et al. 2022). Elevated *ERK1/2* signaling in the absence of *PTPRD* may alter the ability of DA neurons to maintain excitability homeostasis via *D1R*/*ERK*/*KCNQ2* signaling.


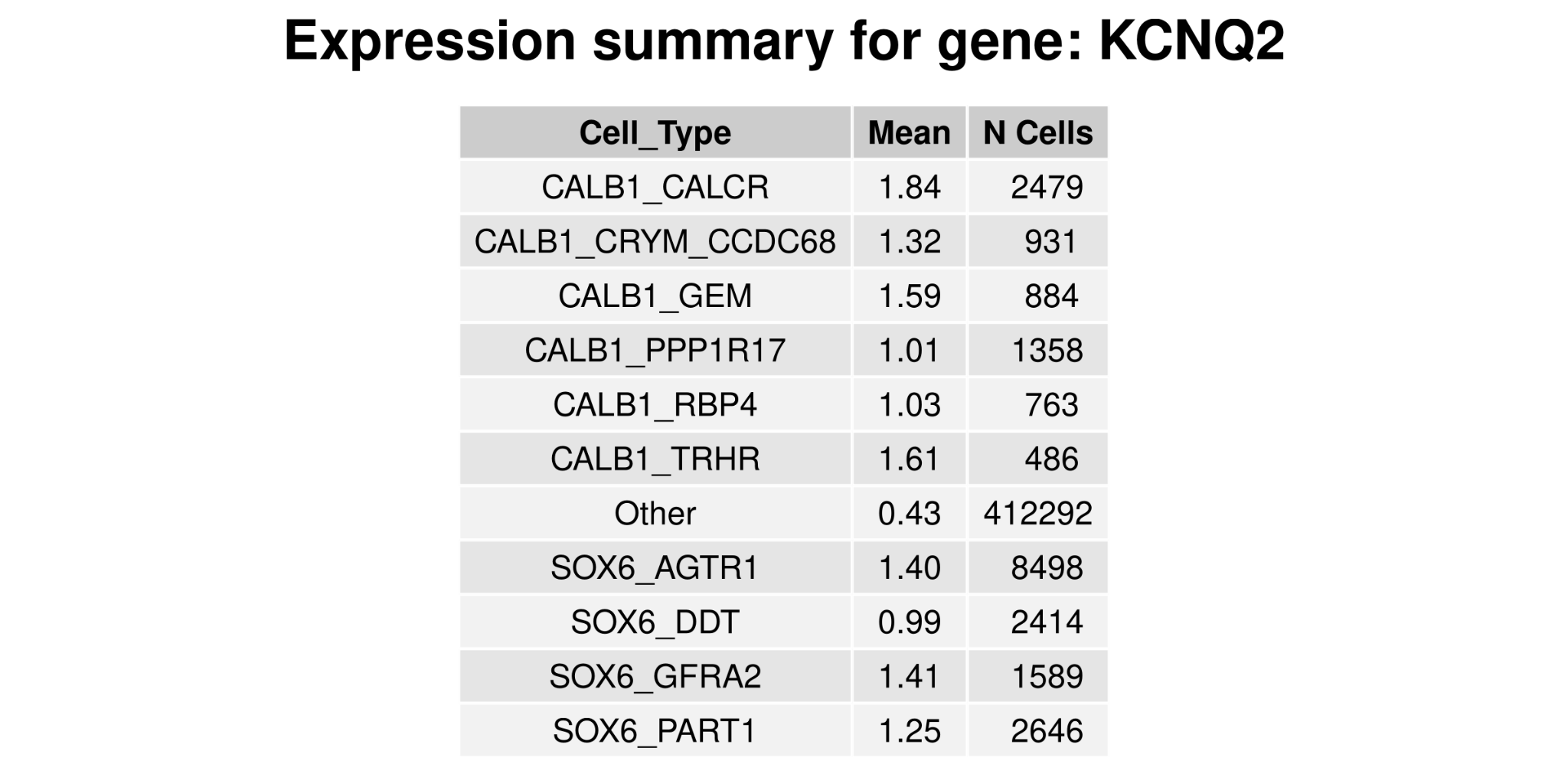


Supplementary Fig 4. RNA expression tables displaying mean UMIs for *KCNQ2* in 10 subclasses of human dopamine neuron clusters from the Kamath et. al 2022 dataset.

**Supplementary Note 6. *BDNF*/*NTRK2*’s role in regulating survival and neuroplasticity**

*TrkB* is implicated in the survival and neuroplasticity of dopamine neurons, specifically as the high affinity, canonical receptor for the neurotrophic factor, brain-derived neurotrophic factor (*BDNF*) [9]. A single injection of *BDNF* into the ventral tegmental area of rats elicits sustained increase in cocaine-seeking behavior lasting up to 30 days, an effect that is negated by the *MEK* inhibitor U0126 [10]. *BDNF* is a promoter of DA neuron survival and axonal growth, regulating survival and neuroplasticity of DA neurons [11].

**Supplementary Note 7. *NTRK2*/*VAV2* and LTP**

Immunoprecipitations with anti-phosphotyrosine antibodies and flag-tagged *TrkB* have supported direct phosphorylation interactions between *VAV2* and *TrkB* in mouse hippocampal neurons. *VAV2* is a guanine exchange factor for the rho family of GTPases, which regulate the actin cytoskeleton, playing an essential role in dendritic spine formation, motility, and morphology [12]. Upon *BDNF* stimulation, reduced increase of *Rac1*-GTP concentrations is observed in *VAV2/3* -/- mice when compared to the wild type, suggesting that *VAV2*’s GEF domain is required for *Rac1*-GTP synthesis. In assays of *BDNF* induced dendritic spine head growth, no significant changes in spine head area were observed in the *VAV2/3* -/- mice, while the WT exhibited significant increases in spine head area. Additionally, post administration of theta-burst stimulation at CA1 glutamatergic synapses, significantly reduced long term potentiation was observed in *VAV2/3* deficient mice when compared to wildtype (Hale et al. 2011). Is it likely that this *VAV2/3* mechanism is a critical component of *Rac1*-GDP’s transition into *Rac1*-GTP in the context of dendritic spine head growth and actin cytoskeleton regulation, which is essential for regulating LTP. In the absence of *PTPRD*, hyperactivation of *TrkB*/*VAV2* signaling may contribute to excessive dendritic spine head growth and chronic excitability of DA neurons.


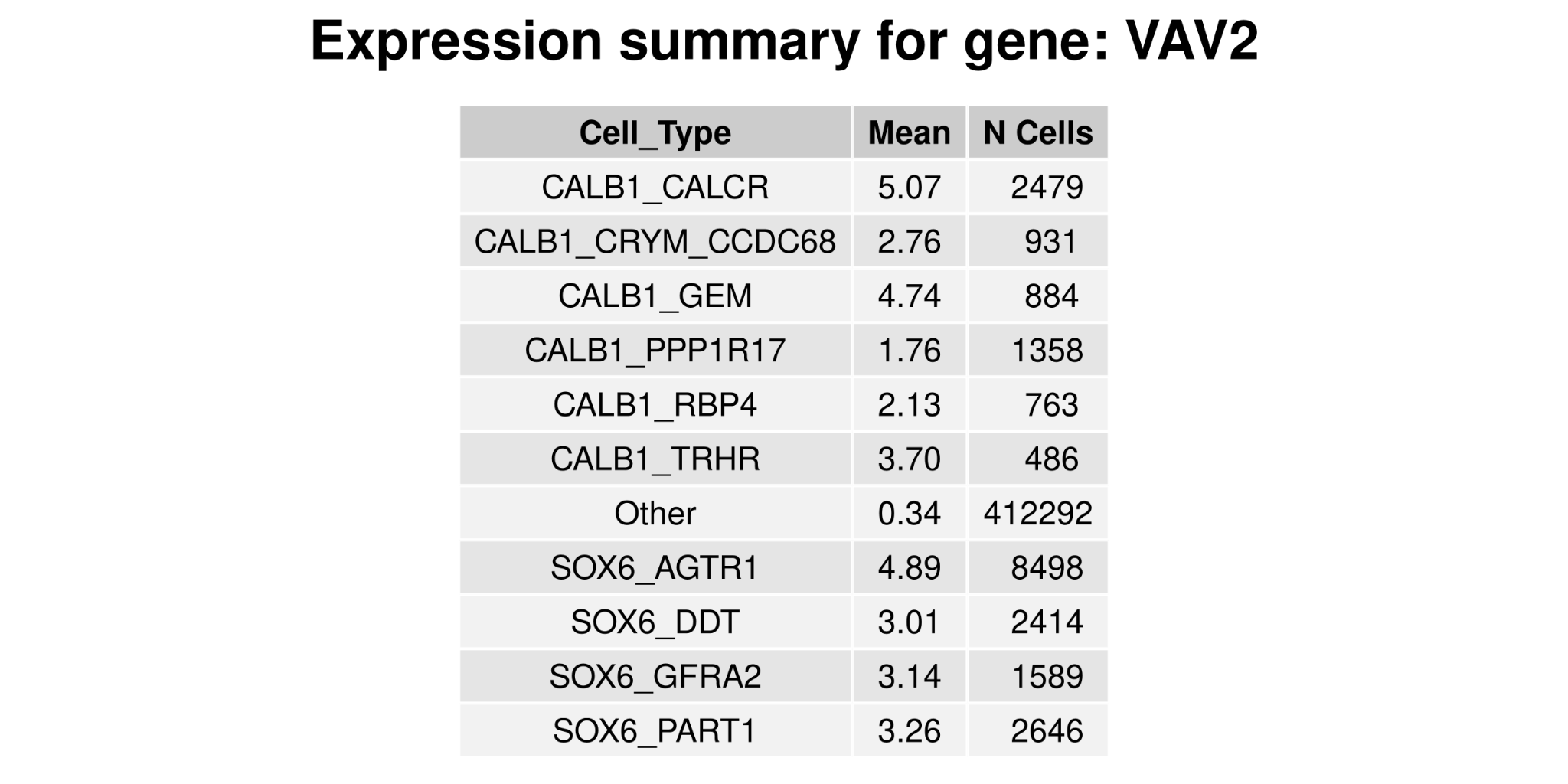


Supplementary Fig 5. RNA expression tables displaying mean UMIs for *VAV2* in 10 subclasses of human dopamine neuron clusters from the Kamath et. al 2022 dataset.


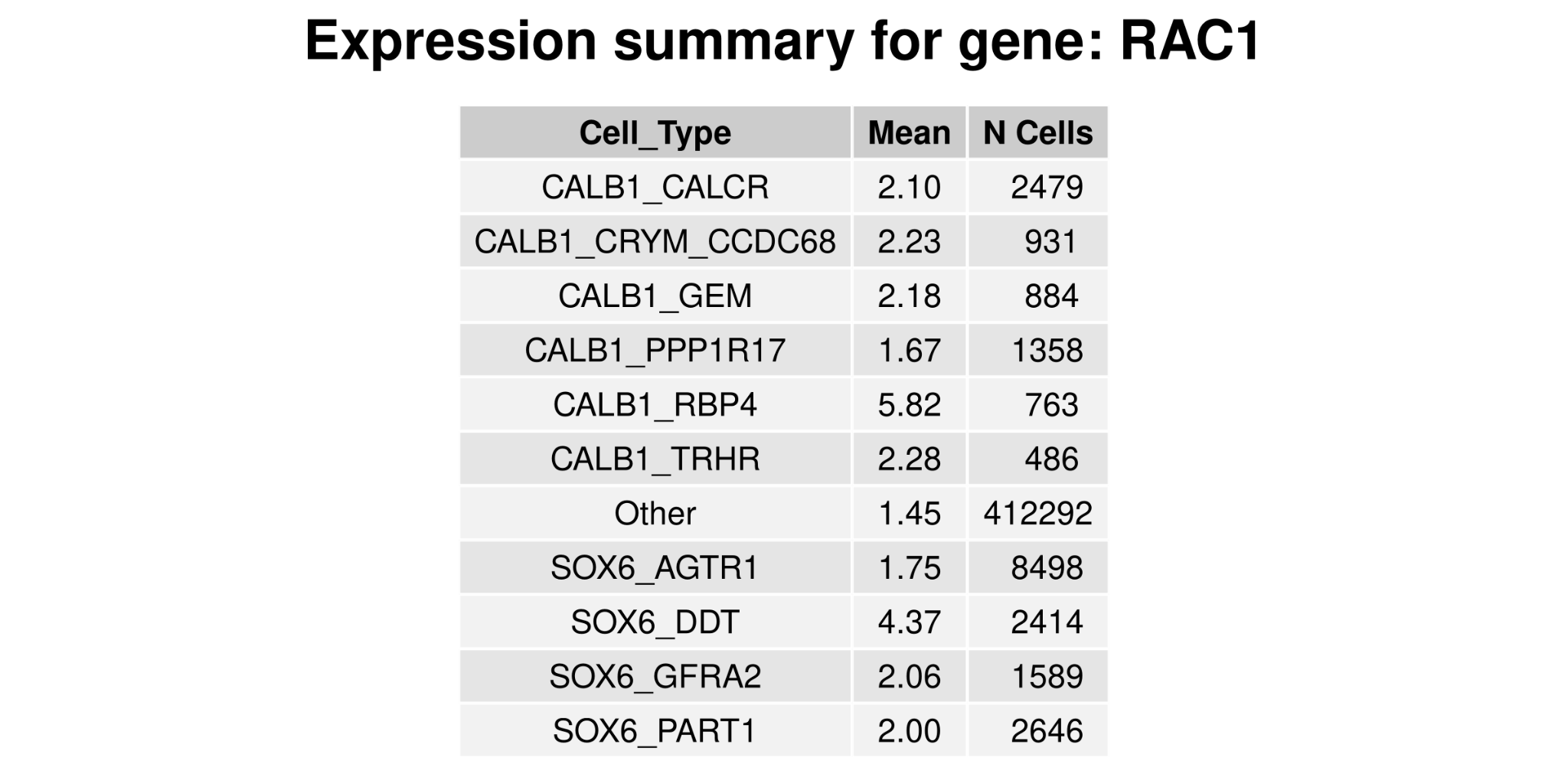
Supplementary Fig 6. RNA expression tables displaying mean UMIs for *RAC1* in 10 subclasses of human dopamine neuron clusters from the Kamath et. al 2022 dataset.

**Supplementary Note 8. *BDNF*/*TrkB* signaling and LTP in DA neurons from glutamatergic synapse input**

Dopamine neurons require *BDNF* to undergo LTP from glutamatergic excitatory synapse input. When presynaptic stimuli of excitatory glutamatergic afferents are applied to dopamine neurons, the production of sustained excitatory postsynaptic potential (EPSP), is only observed when dopamine neurons are pretreated with exogenous *BDNF* in vivo [13]. *BDNF* induced actin cytoskeleton regulation and consequent dendritic spine head growth likely contributes to the observed requirement of *BDNF* for the LTP of DA neurons in response to glutamatergic input across studies.

**Supplementary Note 9. DA neuron *BDNF*/*TrkB* signaling and inhibitory LDP of the GABAergic synapse**

*BDNF* signaling in DA neurons is also implicated in inhibitory long term depression (I-LTD) [14], suggest that *BDNF* acts via *TrkB* to activate phospholipase C and diacylglycerol lipase to cleave phosphatidylinositol 4,5-bisphosphate for the synthesis of 2-arachidonoylglycerol (2-AG), in response to otherwise subthreshold GABAergic afferent stimulation (10 Hz, 5 min). The 2-AG is then released in retrograde fashion by the DA neurons to engage presynaptic cannabinoid receptor type 1 (CB₁) receptors of the GABAergic ISPCs, producing long-term depression of GABAergic IPSC input towards DA neurons. DA-neuron *BDNF* knockout abolishes this eCB-mediated I-LTD, and both bath or systemic application of the *TrkB* agonist *DHF* fully restores it. In further support of this pathway, *BDNF* released onto ventral tegmental area dopamine neurons induced inhibitory LDP [14]. It is likely that *TrkB* signaling in dopamine neurons is required to potentiate inhibitory LTP upon stimulation from GABAergic synaptic currents. The absence of *PTPRD* may contribute to excessive retrograde inhibition of GABAergic synapses via hyperactivated *TrkB* signaling, contributing to the chronic excitation of DA neurons.

**Supplementary Note 10. *PTPRD* deficient mice experience enhanced long-term potentiation**

A common trend we have identified from the potential downstream effectors of *PTPRD* is chronic excitability. These findings are consistent with *PTPRD* KO/Inhibition literature that assay neuronal excitability, of which there is a singular publication. Uetani et al. 2000 [15], generated *PTPRD*-deficient mice and found that, despite normal hippocampal histology, these animals exhibited severe growth retardation and semi-lethality due to impaired food intake. Although confirmatory research would be required, the absence of *PTPRD*’s D1 phosphatase activity in DA neuron-mediated reward circuitry may have contributed to decreased food intake. Behavioral assays revealed that *PTPRD*-deficient mice had significant deficits in spatial learning and working memory. Long-term potentiation at both CA1 and CA3 synapses was significantly augmented in *PTPRD*-deficient mice. These findings imply *PTPRD* as a critical regulator of hippocampal synaptic neuroplasticity and underscore that optimal, not maximal, LTP is required for normal memory formation [15]. With the lack of research surrounding *PTPRD* assays in DA neurons, we are left to extrapolate from research of other neurotransmitter specific neurons. As the current literature suggests, core pathways like *TrkB*/*BDNF* and *MEK*/*ERK* are typically conserved across neurons [16] [17]. It is biologically plausible that *PTPRD* may play non-redundant roles in DA neuron LTP, from which strict homeostatic control is required to achieve optimal reward processing.

**Supplementary Note 11. Sustained excitability and its effects on metaplasticity**

When neurons are chronically excited, the signal threshold for inducing LTP increases, a phenomenon known as metaplasticity. The chronic excitation of hippocampal CA1 neurons triggers homeostatic adaptations that raise the threshold for structural LTP (sLTP) [18]. After 24 hours of bicuculline treatment, a GABA receptor antagonist, NMDAR‐mediated Ca²⁺ influx was downregulated and Calcium/calmodulin-dependent protein kinase II (*CaMKII*) was inhibited, a protein that plays essential roles in driving dendritic spine growth. As a result, the same glutamate uncaging or photoactivatable *CaMKII* activation that normally induces sLTP in controls fails entirely unless much stronger stimulation is administered. (Ueda et al. 2000). Chronic overexcitation of neurons moves the LTP signal threshold upward, preventing hyperactive circuits. The phenomenon of metaplasticity may underlie PTPRD-deficient mice’s altered behavioral responses to cocaine administration, especially the reduction in break points and lever presses. The absence of *PTPRD* and hyperactivation of *TrkB* signaling may contribute to chronic excitation, increasing LTP signal threshold and attenuating cocaine’s ability to induce LTP in dopamine neurons and reinforce reward-related behavior.


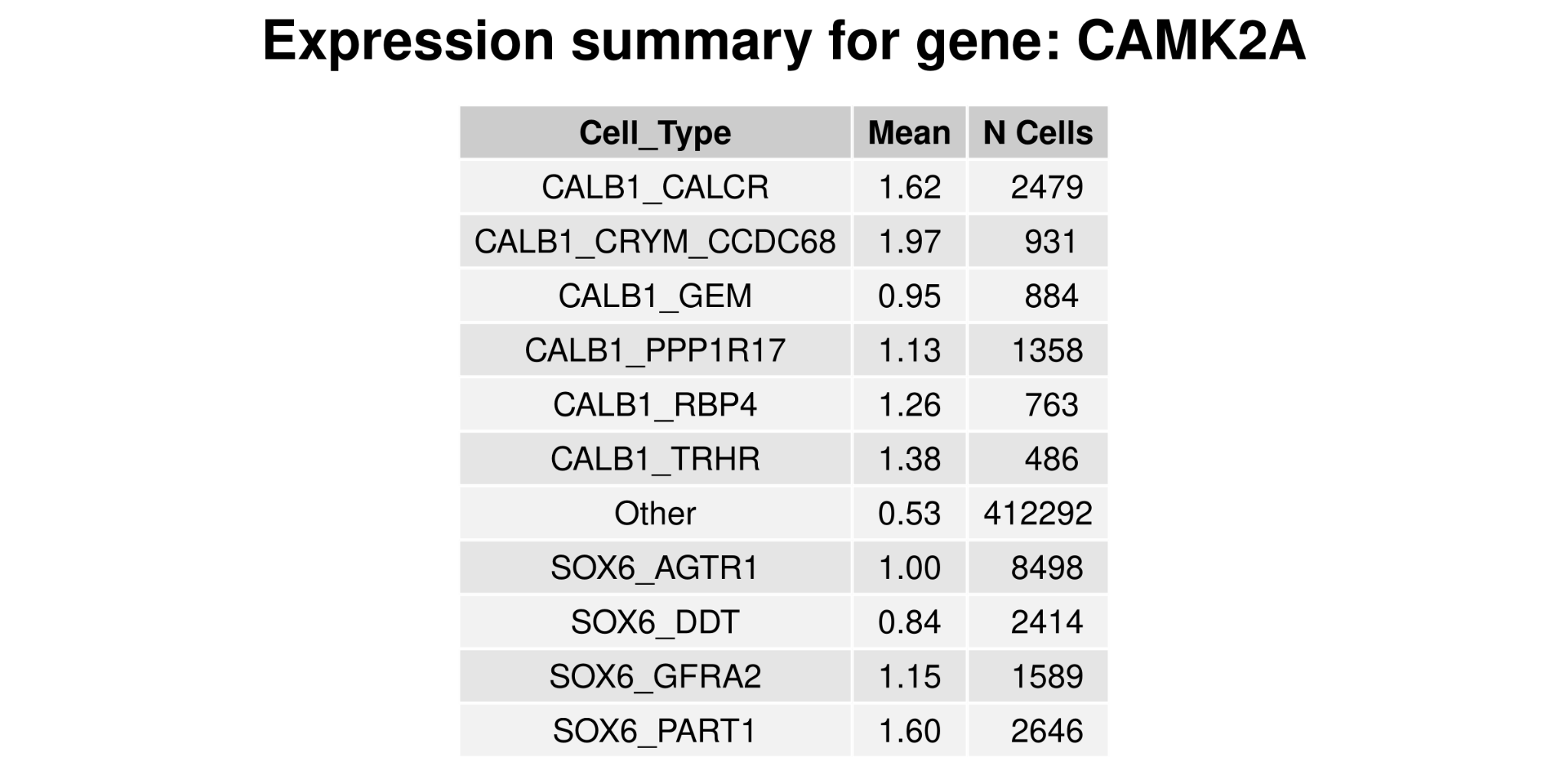


Supplementary Fig 7. RNA expression tables displaying mean UMIs for *CAMK2A* in 10 subclasses of human dopamine neuron clusters from the Kamath et. al 2022 dataset.


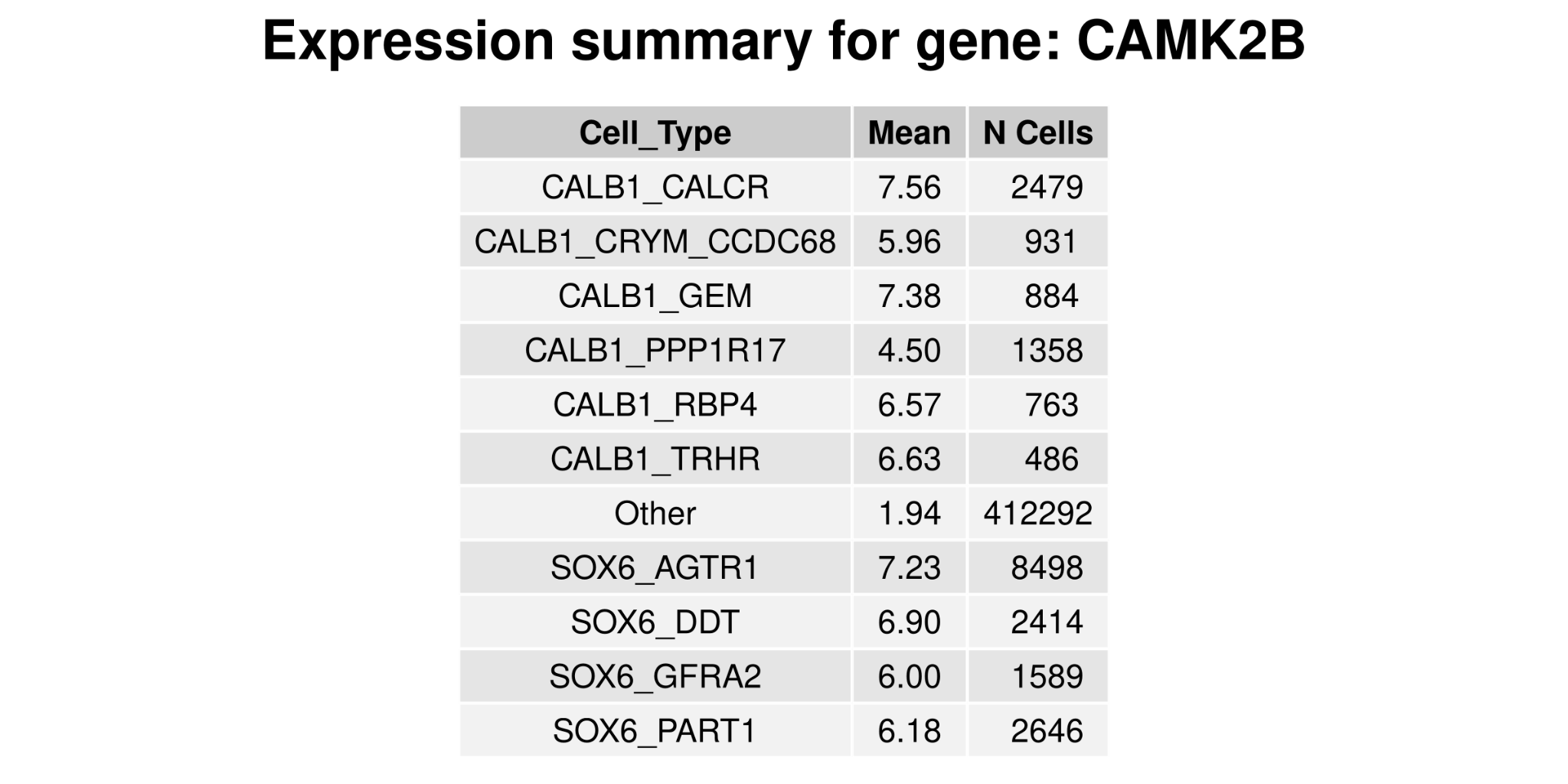


Supplementary Fig 8. RNA expression tables displaying mean UMIs for *CAMK2B* in 10 subclasses of human dopamine neuron clusters from the Kamath et. al 2022 dataset.


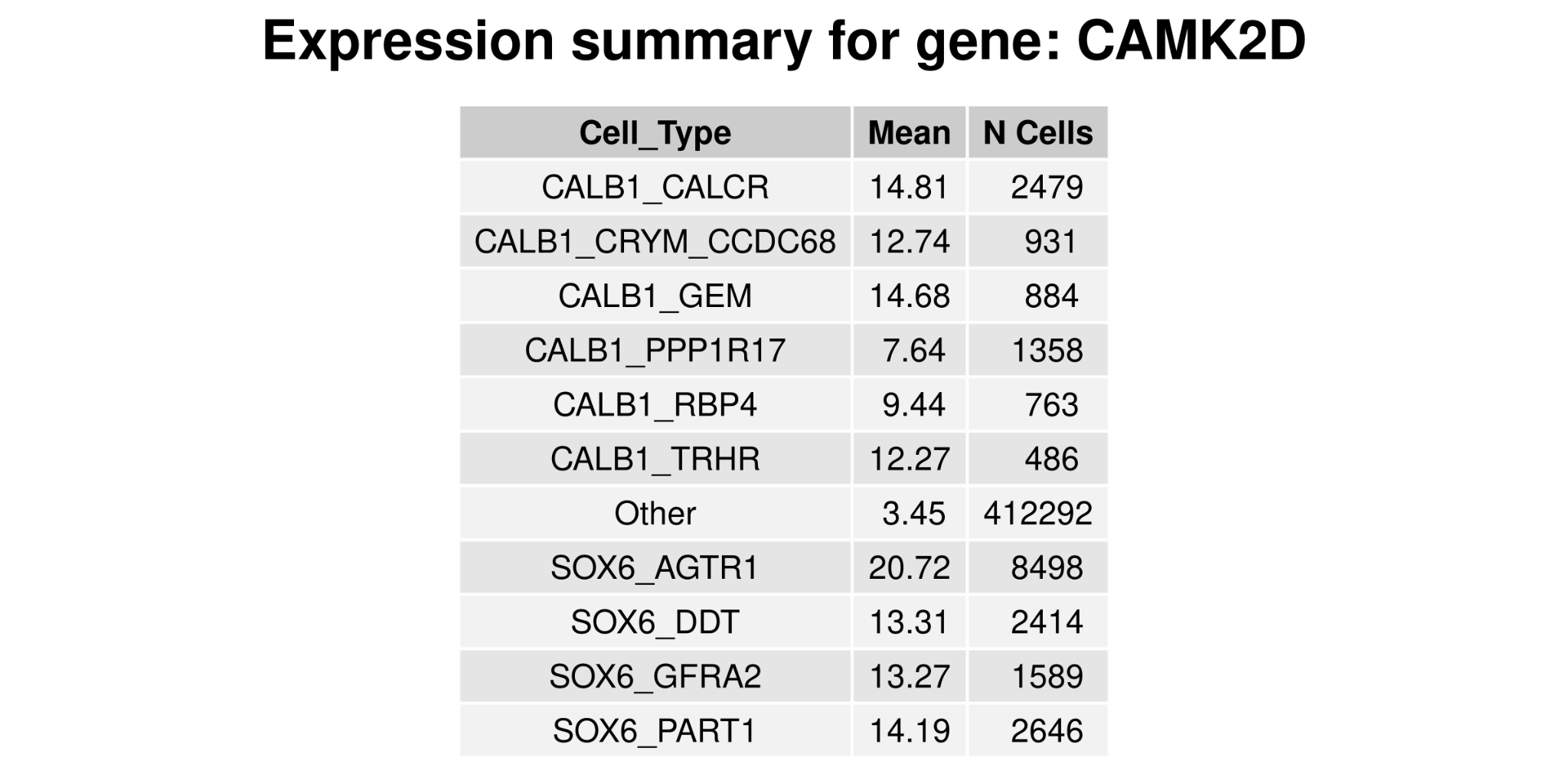


Supplementary Fig 9. RNA expression tables displaying mean UMIs for *CAMK2D* in 10 subclasses of human dopamine neuron clusters from the Kamath et. al 2022 dataset.


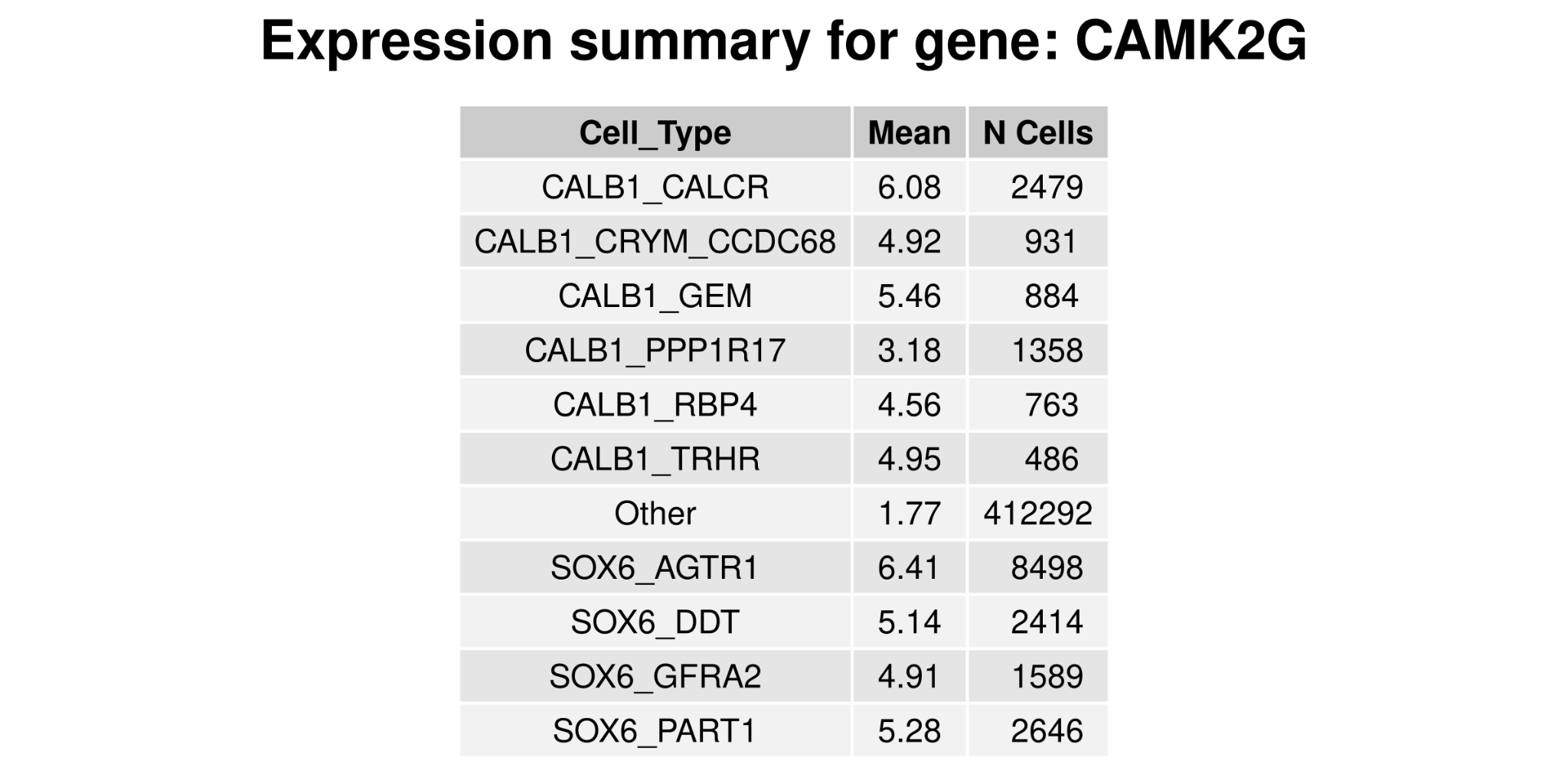


Supplementary Fig 10. RNA expression tables displaying mean UMIs for *CAMK2G* in 10 subclasses of human dopamine neuron clusters from the Kamath et. al 2022 dataset.

**Supplementary Note 12. *VAV2* and *DAT* transporter trafficking in the mesolimbic pathway**

The literature supports that LAR-family RPTPs dephosphorylate tyrosine residues involved in *RET* activation, as well as prevent dimerization to ultimately hinder autophosphorylation. These interactions are independent of glial cell-line derived neurotrophic factor (*GDNF*), *RET*’s canonical ligand, and serve to downregulate *RET* signaling pathways [15] [19]. The loss of *PTPRD* may contribute to hyperactivated RET signaling. *GDNF* stimulation induces Tyr-905 phosphorylation of *RET*, which is accompanied by a rise in *VAV2* tyrosine phosphorylation [20]. Interestingly, *VAV2* is an essential mediator of *DAT* transporter membrane trafficking for DA neurons in the ventral tegmental area [20]. *VAV2* gene knockdown mice experienced increased levels of surface *DAT*, leading to significantly increased concentrations of intracellular DA in the nucleus accumbens. Consequently, the mutants exhibited lower cocaine-induced locomotor activation, a behavioral response highly correlated with the capacity to rapidly upregulate *DAT* upon cocaine administration [21]. When compared to the WT, *VAV2*-deficient mice exhibited elevated surface *DAT* in the nucleus accumbens after saline solution exposure, and exhibited decreased surface *DAT* after cocaine administration. The researchers did not experiment with *VAV2* overexpressive mice, however surface *DAT* was measured in culture with plasmid transfections of *VAV2* and constitutively activated *VAV2* HEK293 cells engineered to stably overexpress *DAT*. Western blots displayed significantly less surface *DAT* in the *VAV2* over-expressive and constitutively activated *VAV2* HEK293 cultures [20]. These findings are suggestive of *VAV2*’s role in the bidirectional trafficking of *DAT*. *TrkB* hyperexcitability and consequent increases in *RET* and *VAV2* tyrosine phosphorylation in the absence of *PTPRD* may contribute to increased *VAV2*/*RET* mediated endocytosis of *DAT*, which may underlie the observed behavioral responses to cocaine in *PTPRD* KO/inhibition mice.


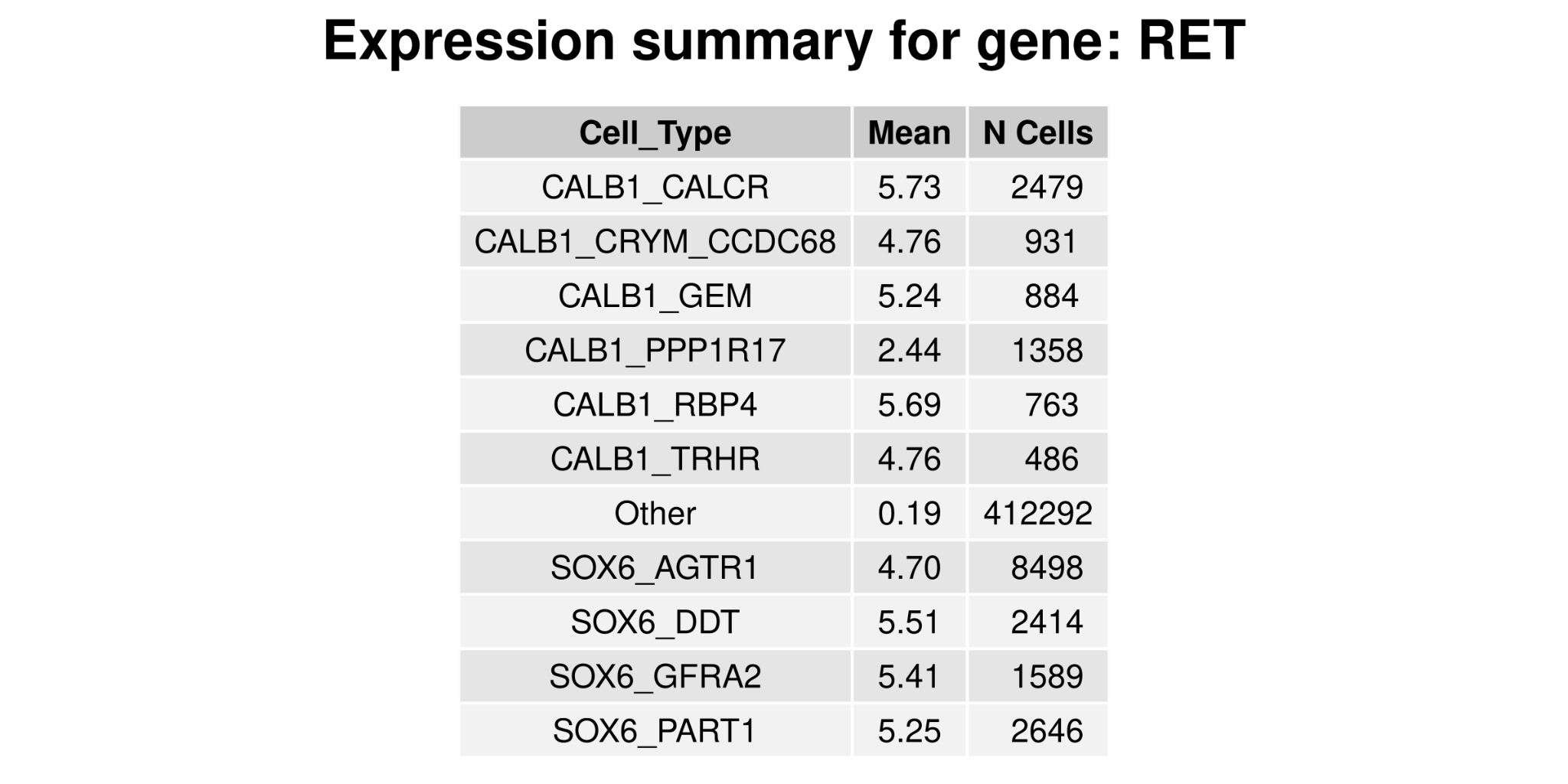


Supplementary Fig 11. RNA expression tables displaying mean UMIs for *RET* in 10 subclasses of human dopamine neuron clusters from the Kamath et. al 2022 dataset.

**Supplementary Note 13. *ERK1/2* and *DAT* transporter**

*ERK1/2* regulates dopamine transporter surface expression and transport capacity (Moron et al. 2003). The inactivation of *ERK1/2* in DA neurons results in an increase of surface *DAT* transporter and a reduction in *DAT* phosphorylation, leading to decreased DA under the curve (Bernstein et al. 2024). Mice that received a Cre-dependent AAV to overexpress mitogen-activated protein kinase 3 and inhibit *ERK1/2* signaling in VTA dopaminergic neurons exhibited significant reduction in motivation for cocaine with less breakpoints and lever presses than the wildtype. However, *ERK1/2* inactivation did not alter cocaine self-administration and rate of cocaine intake [22]. In the context of *PTPRD* KO/inhibition, it is important to note that the literature is mixed in supporting elevated phosphorylation of *ERK1/2*. Conditional knockouts of *PTPRD* in *EMX1*+ neural progenitor cells only increased phosphorylation of *MEK1/2* in mice, whereas global mice knockouts of PTPRD increased both phosphorylation of *MEK1/2* and *ERK1/2* [23]. Further research would be necessary to verify if PTPRD inhibition in DA neurons results in altered ERK1/2 activity. However, it is plausible that PTPRD inhibition in DA neurons hyperactivates ERK1/2 signaling, resulting in chronic excitability from excessive deactivation of KCNQ2 ion channels and aberrant homeostatic control of ERK1/2-mediated surface DAT expression and phosphorylation.

**Supplementary Note 14. *PKC* signaling and *DAT***

The literature is consistent in supporting protein kinase C (*PKC*) as a downstream effector of *BDNF* signaling [24]. Interestingly, *PKC* is heavily implicated in *DAT* transporter trafficking [25]. Sustained activation of *PKC*, typically by phorbol esters, drives rapid, clathrin and dynamin-dependent internalization of *DAT* into endosomes and lysosomes, lowering surface *DAT* and DA uptake capacity [26] [27]. *PKC* inhibitors block this phorbol ester–induced DAT loss [26]. PKC encompasses bidirectional *DAT* transporter trafficking regulation, a very brief (≤ 90 s) amphetamine administration selectively recruits *PKCβ* to drive rapid DAT exocytosis to the plasma membrane, boosting surface DAT *and* amphetamine-stimulated DA efflux from the synapse [28] [29]. During prolonged amphetamine exposure (60 min), WT synaptosomes revert to *DAT* internalization, whereas *PKCβ*-deficient mice show delayed *DAT* insertion at 60 min, demonstrating that *PKCβ* is involved in both the timing and directionality of *DAT* trafficking [28]. The absence of *PTPRD* may contribute to the hyperactivation of *PKC* signaling, attenuating the ability of DA neurons to maintain homeostatic control of *DAT* surface levels.


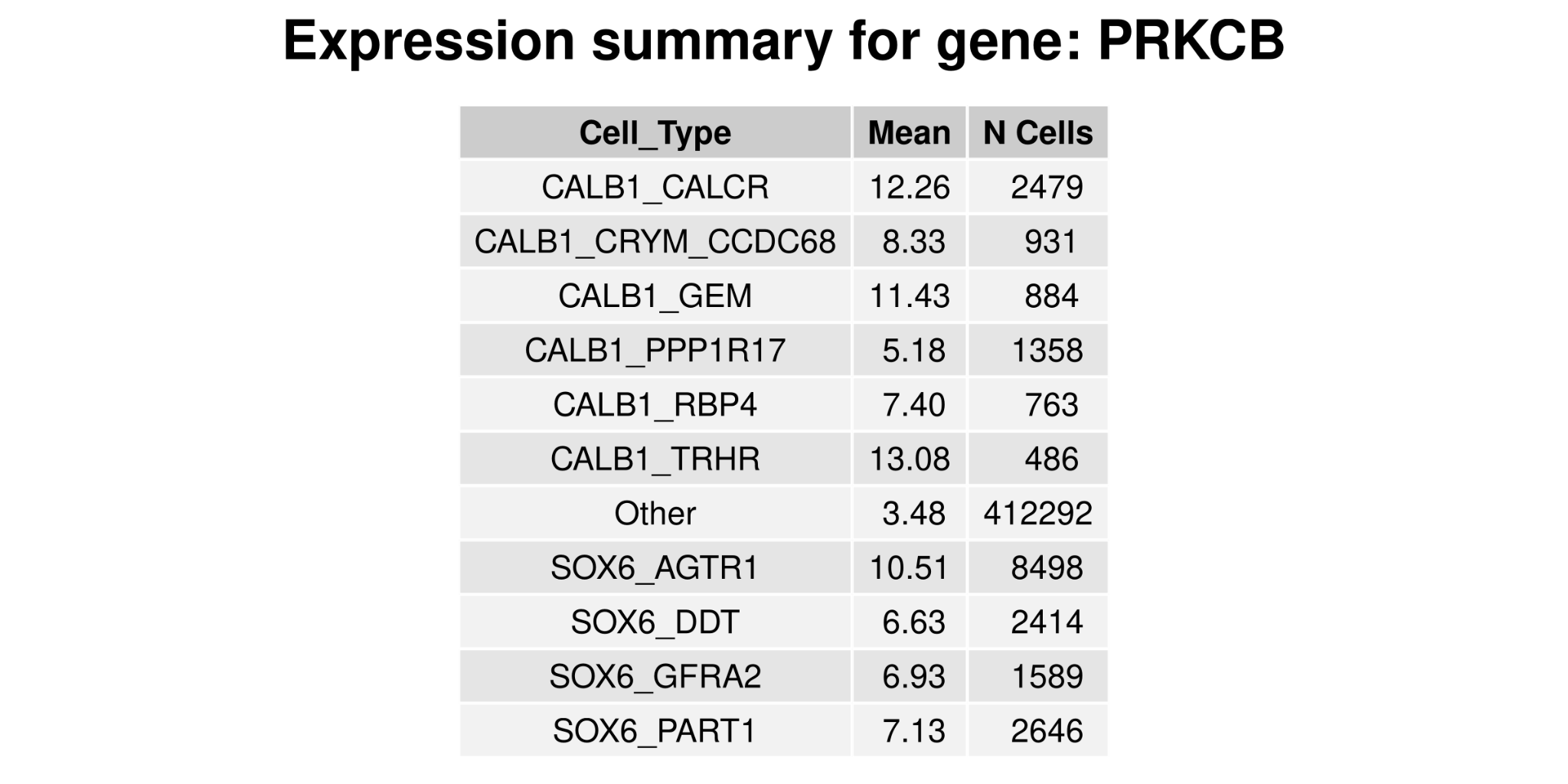
Supplementary Fig 12. RNA expression tables displaying mean UMIs for *PRKCB* in 10 subclasses of human dopamine neuron clusters from the Kamath et. al 2022 dataset.

**Supplementary Note 15. *PTPRD*-F1 model proposal**

Collectively, the literature combined with *PTPRD’s* significance for F1 in the DA neuron H-MAGMA analysis speculate *PTPRD* indirectly regulates dopaminergic neuroplasticity. In the DA neuron H-MAGMA analysis, of the statistically significant GWAS SNPs mapped to PTPRD (p < 5e-08), all were either intronic or intergenic. No missense, frameshift, or nonsense mutation SNPs passed significance thresholds, suggesting that the differential expression of *PTPRD* contributes to the development of ADHD, MDD, and BPD as opposed to changes in amino acid sequence. Loss of *PTPRD*’s D1 phosphatase domain may permit the hyperactivation of *TrkB* and *RET* signaling pathways, contributing to chronic excitation and aberrant DAT-mediated uptake of synaptic dopamine. Hyperactivated *TrkB* and *RET* signaling may increase *ERK1/2*, *PKC*, and *VAV2* phosphorylation and activity. The elevated occurrence of downstream *VAV2* mediated dendritic spine head growth may attenuate homeostatic control of structural LTP that respond to glutamatergic input. The elevated downstream occurrence of phosphorylated *ERK1/2* may attenuate homeostatic control of both *DAT* transporter surface expression and *KCNQ2* K+ ion channels. The elevated occurrence of downstream *PKC* and *VAV2* activation may attenuate homeostatic control of bidirectional *DAT* transporter surface expression. In addition, excessive *TrkB* signaling may induce elevated retrograde inhibition of inhibitory GABAergic synapses (Inhibitory LTP). The coalescence of these *PTPRD* risk factors may contribute to the chronic excitation of DA neurons through increases of structural and inhibitory LTP and decreased *DAT*-mediated synaptic dopamine uptake. These factors may alter neuroplasticity and metaplasticity in DA neurons, increasing the signaling threshold to induce optimal potentiation of both structural and inhibitory LTP and DA-mediated signal transduction between dopaminergic neurons to maintain reward-associated circuit homeostasis. We propose that these mechanisms may help explain the consistent trend of altered behavioral responses to cocaine in *PTPRD*-deficient mice, especially in regards to decreased motivation for cocaine.

**Supplementary Note 16. Exploratory, hypothesis-generating *PTPRD* fine-mapping using 1000 Genomes external reference panel and F1 summary statistics**

The H-MAGMA DA neuron variant annotation mapped SNPs to *PTPRD* from 7.4 Mb to 12 Mb on chromosome 9, suggestive of cis-regulatory elements (We will refer to this region as *PTPRD* extended). In attempts to explore the gene’s histology and encompass any potential regulatory features, we used the same bounds to extend our lens of *PTPRD* past its hg19 standardized coordinates on chromosome 9, 8314246-10612723. We then estimated causal SNP posterior inclusion probabilities and local heritability for F1 in regions of interest, as well as analyzed the LD structure of *PTPRD* extended (Supplementary Fig 13) (Supplementary Table 32). There is a significant downstream spike in LD from about 10.9Mb to 12Mb on chromosome 9 (Supplementary Figure 13). Functionally relevant domains such as expression quantitative trait loci (eQTLs), methylation quantitative trait loci (meQTLs), and chromatin loops exhibit reduced recombination [30]. This region may host a plethora of regulatory elements. However, it is important to note that the use of external reference panels with small sample size that lack in-sample genotypes, such as the 1000 Genomes Project we utilize, introduce bias and unstable estimates. We acknowledge that LD estimates and causal posterior inclusion probabilities may be noisy; results should therefore be interpreted with caution.


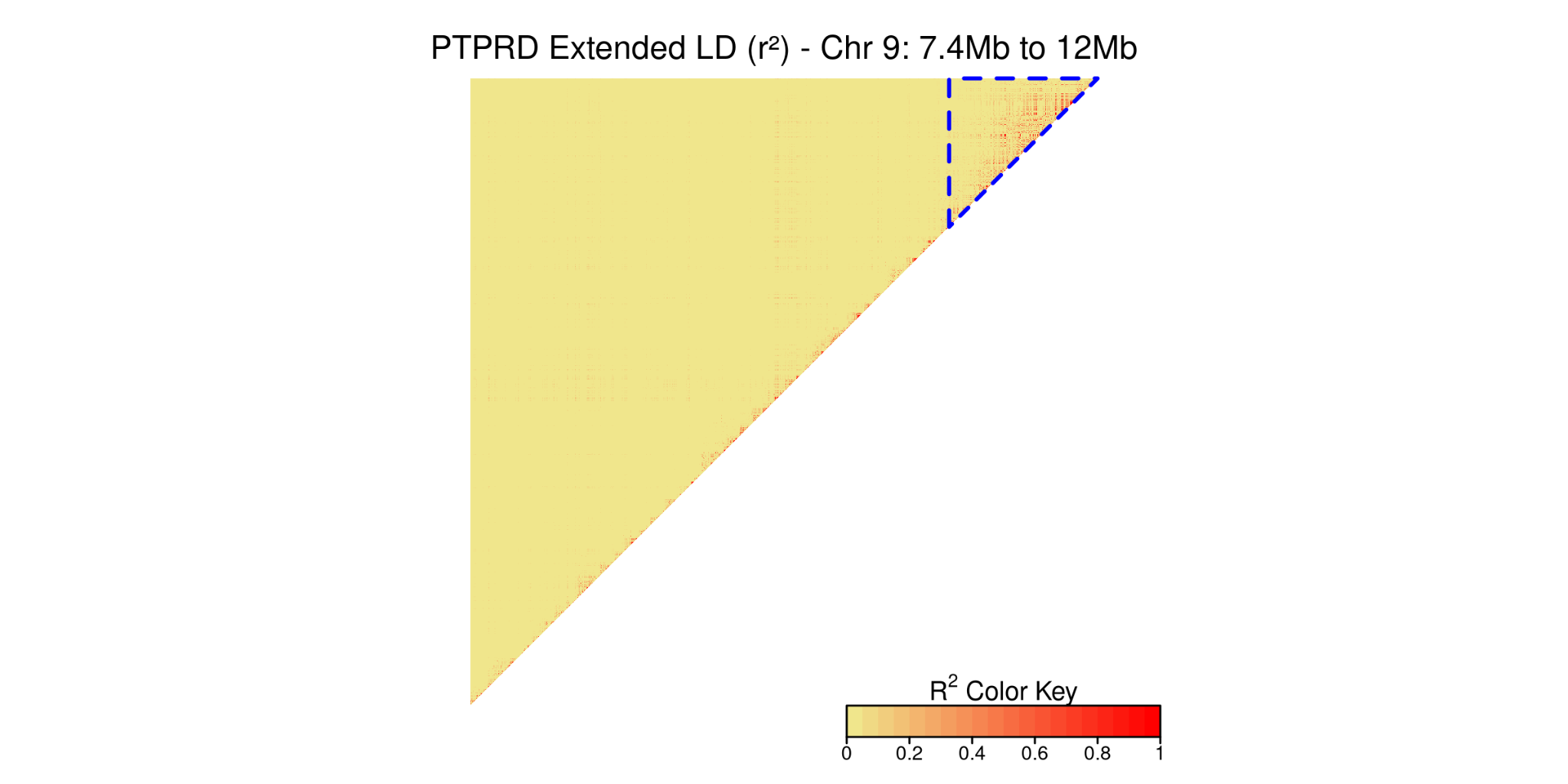


Supplementary Fig 13. Linkage disequilibrium heatmap of *PTPRD* extended (Chr 9: 7.4Mb to 12Mb), consisting of the top off-diagonals of an r^2^ matrix calculated using the 1000 Genomes Project EUR reference panel. The blue box highlights the aforementioned recombination valley (Chr 9, 10.9Mb to 12 Mb).

Concurrently, there is an F1 risk locus within this elevated region of LD just a few hundred kilobases downstream of the hg19 standardized *PTPRD* coordinates. This locus encompasses 574 F1 significant SNPs (p < 5e-08), and 3 F1 index SNPs (r^2^ < 0.1 and p < 5e-08). Using LAVA, we estimated local genetic heritability using the F1 summary statistics as input [31]. The observed local SNP heritability for the elevated region of LD from 10.9Mb to 12Mb on chromosome 9 was 1.17e-04 (p-value = 9.29e-07), and for PTPRD extended the observed local SNP heritability was 5.66e-04 (p-value = 4.2e-35). The estimates suggest that these regions harbor significant F1 SNP heritability, magnitudes more than would what be expected by chance. To define a risk locus for further analysis in the context of *PTPRD* fine-mapping, we added 100 Kb to both the most upstream F1 index SNP and to the most downstream SNP mapped to *PTPRD* from the midbrain dopaminergic H-MAGMA annotation in attempts to encapsulate a larger percentage of the F1 risk locus’s regulatory features (Fig 19). We then utilized mixer-finemap to estimate causal posterior inclusion probabilities for each SNP in the risk locus (Akdeniz et al. 2024). Given the concurrence of abnormally high SNP heritability and reduced recombination, this region may be a DA neuron regulatory domain, housing cis-eQTLs for *PTPRD* and a plethora of other genes.
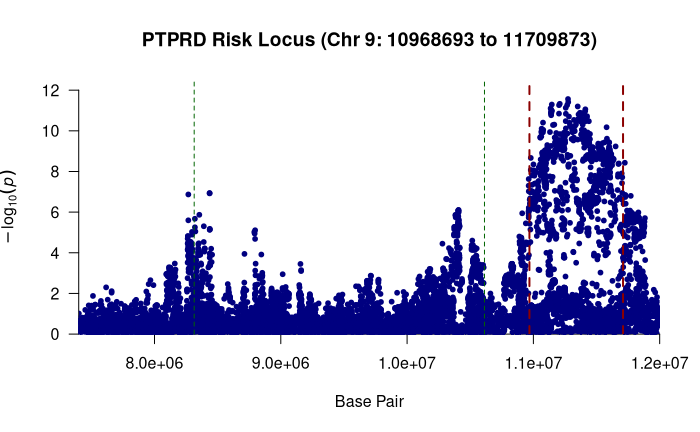


Supplementary Fig 14. F1 Manhattan plot of *PTPRD* extended with the *PTPRD* risk locus bounds denoted by the red dotted lines and standardized hg19 PTPRD coordinates denoted by the green dotted lines.


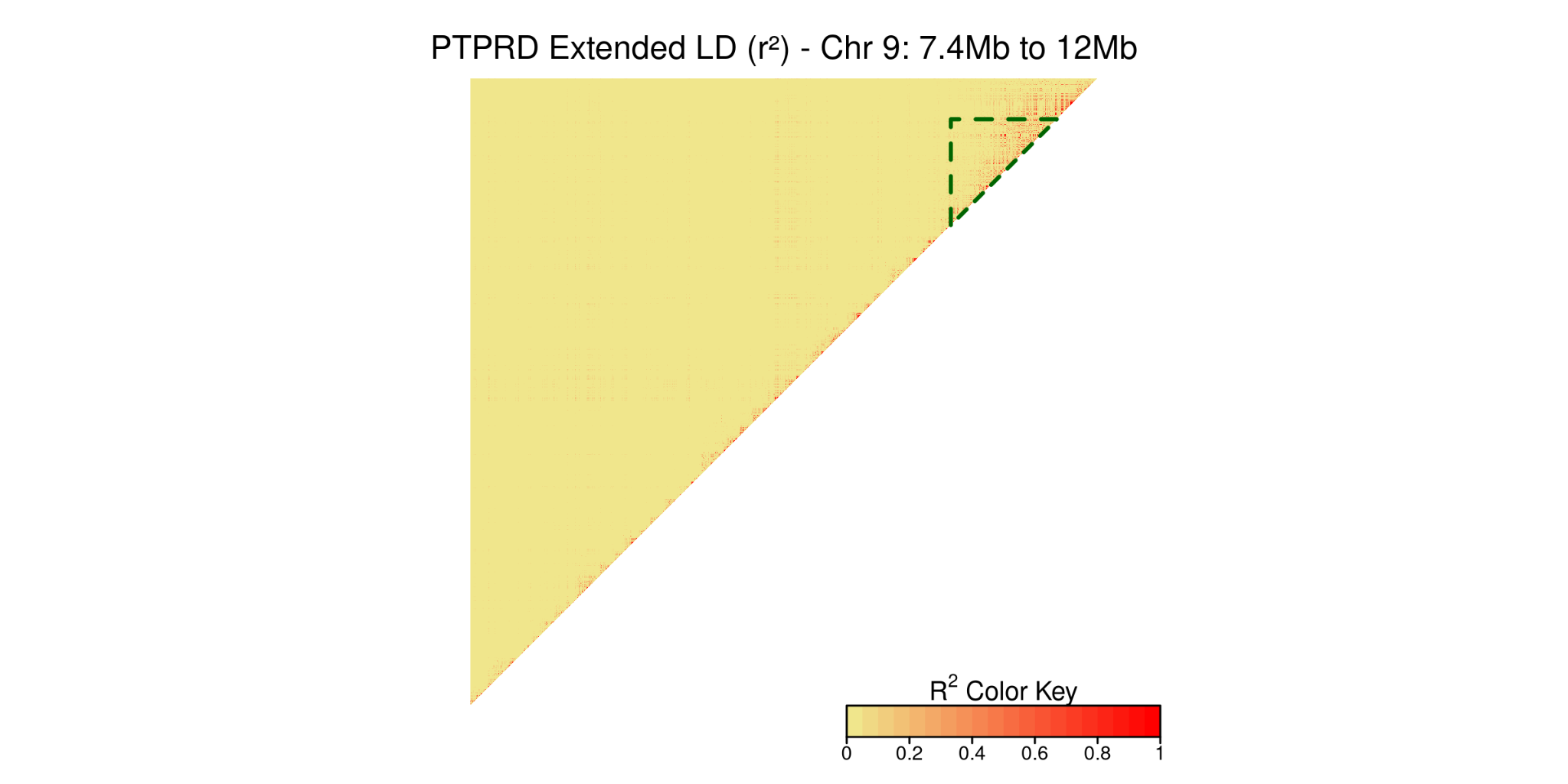


Supplementary Fig 15. Linkage disequilibrium heatmap of *PTPRD* extended with the *PTPRD* risk locus highlighted by the green dotted line (Chr 9, 10968693 to 11709873), consisting of the top off-diagonals of an r^2^ matrix calculated using the 1000 Genomes Project EUR reference panel.


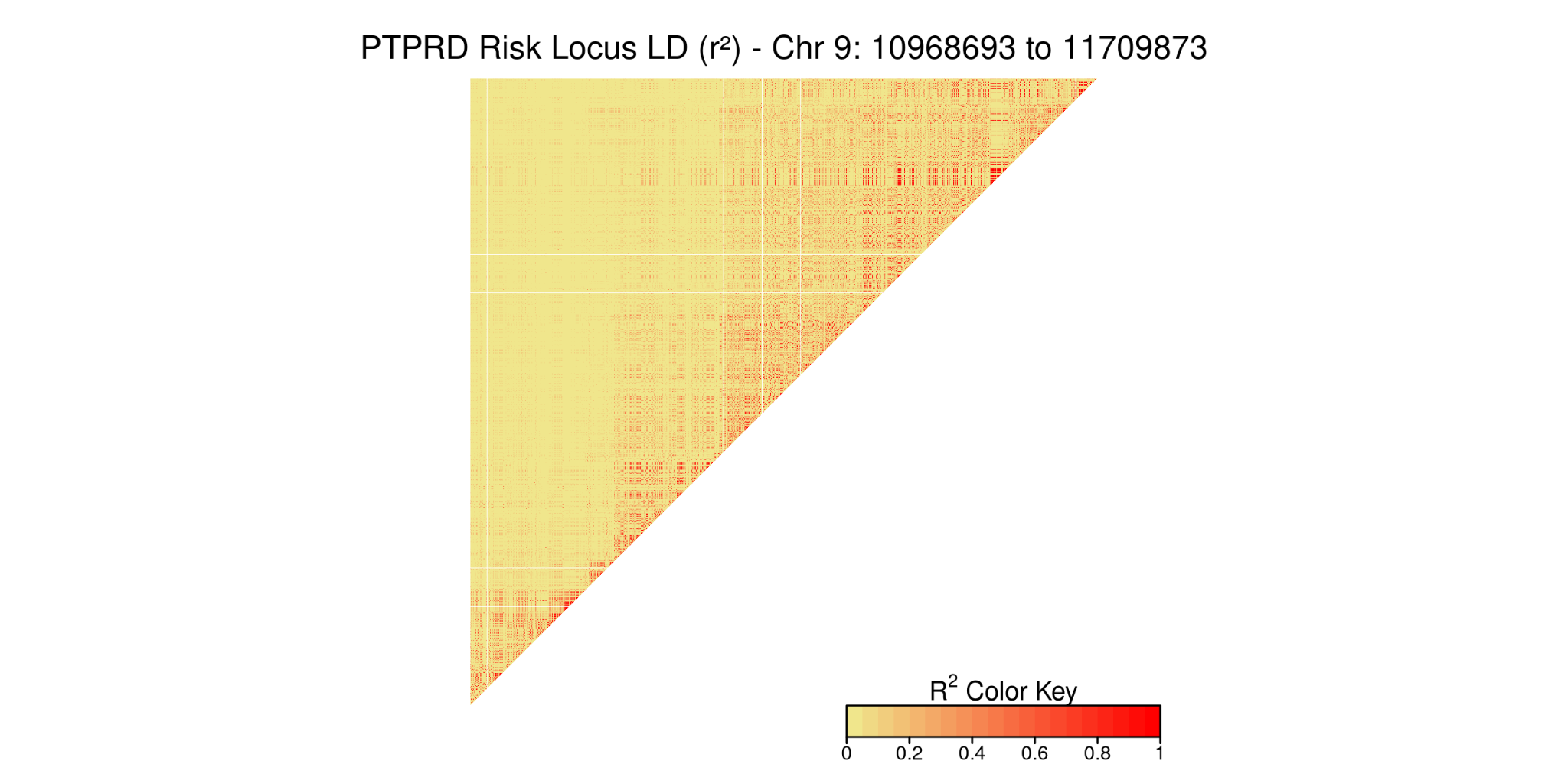


Supplementary Fig 16. Linkage disequilibrium heatmap of PTPRD risk locus), consisting of the top off-diagonals of an r^2^ matrix calculated using the 1000 Genomes Project EUR reference panel.

**References**

1. Takei Y, Zheng S, Yun J, Shah S, Pierson N, White J, et al. Single-cell nuclear architecture across cell types in the mouse brain. Science. 2021;374: 586–594. doi:10.1126/science.abj1966

2. Liu S, Wang CY, Zheng P, Jia BB, Zemke NR, Ren P, et al. Cell type–specific 3D-genome organization and transcription regulation in the brain. Sci Adv. 11: eadv2067. doi:10.1126/sciadv.adv2067

3. Woodward DJ, Thorp JG, Middeldorp CM, Akóṣílè W, Derks EM, Gerring ZF. Leveraging pleiotropy for the improved treatment of psychiatric disorders. Mol Psychiatry. 2025;30: 705–721. doi:10.1038/s41380-024-02771-7

4. Emperador-Melero J, de Nola G, Kaeser PS. Intact synapse structure and function after combined knockout of PTPδ, PTPσ, and LAR. eLife. 10: e66638. doi:10.7554/eLife.66638

5. Han KA, Lee H-Y, Lim D, Shin J, Yoon TH, Liu X, et al. Receptor protein tyrosine phosphatase delta is not essential for synapse maintenance or transmission at hippocampal synapses. Mol Brain. 2020;13: 94. doi:10.1186/s13041-020-00629-x

6. Uhl GR, Martinez MJ. PTPRD: neurobiology, genetics, and initial pharmacology of a pleiotropic contributor to brain phenotypes. Ann N Y Acad Sci. 2019;1451: 112–129. doi:10.1111/nyas.14002

7. Kamath T, Abdulraouf A, Burris SJ, Langlieb J, Gazestani V, Nadaf NM, et al. Single-cell genomic profiling of human dopamine neurons identifies a population that selectively degenerates in Parkinson’s disease. Nat Neurosci. 2022;25: 588–595. doi:10.1038/s41593-022-01061-1

8. Nagai T, Nakamuta S, Kuroda K, Nakauchi S, Nishioka T, Takano T, et al. Phosphoproteomics of the Dopamine Pathway Enables Discovery of Rap1 Activation as a Reward Signal In Vivo. Neuron. 2016;89: 550–565. doi:10.1016/j.neuron.2015.12.019

9. Hyman C, Hofer M, Barde Y-A, Juhasz M, Yancopoulos GD, Squinto SP, et al. BDNF is a neurotrophic factor for dopaminergic neurons of the substantia nigra. Nature. 1991;350: 230–232. doi:10.1038/350230a0

10. Lu L, Dempsey J, Liu SY, Bossert JM, Shaham Y. A Single Infusion of Brain-Derived Neurotrophic Factor into the Ventral Tegmental Area Induces Long-Lasting Potentiation of Cocaine Seeking after Withdrawal. J Neurosci. 2004;24: 1604–1611. doi:10.1523/JNEUROSCI.5124-03.2004

11. Østergaard K, Jones SA, Hyman C, Zimmer J. Effects of Donor Age and Brain-Derived Neurotrophic Factor on the Survival of Dopaminergic Neurons and Axonal Growth in Postnatal Rat Nigrostriatal Cocultures. Exp Neurol. 1996;142: 340–350. doi:10.1006/exnr.1996.0203

12. Liu BP, Burridge K. Vav2 Activates Rac1, Cdc42, and RhoA Downstream from Growth Factor Receptors but Not β1 Integrins. Mol Cell Biol. 2000;20: 7160–7169. Available: https://www.ncbi.nlm.nih.gov/pmc/articles/PMC86269/

13. Pu L, Liu Q, Poo M. BDNF-dependent synaptic sensitization in midbrain dopamine neurons after cocaine withdrawal. Nat Neurosci. 2006;9: 605–607. doi:10.1038/nn1687

14. Zhong P, Liu Y, Hu Y, Wang T, Zhao Y, Liu Q. BDNF Interacts with Endocannabinoids to Regulate Cocaine-Induced Synaptic Plasticity in Mouse Midbrain Dopamine Neurons. J Neurosci. 2015;35: 4469–4481. doi:10.1523/JNEUROSCI.2924-14.2015

15. Uetani N, Bertozzi K, Chagnon MJ, Hendriks W, Tremblay ML, Bouchard M. Maturation of ureter-bladder connection in mice is controlled by LAR family receptor protein tyrosine phosphatases. J Clin Invest. 2009;119: 924–935. doi:10.1172/JCI37196

16. Gupta VK, You Y, Gupta VB, Klistorner A, Graham SL. TrkB Receptor Signalling: Implications in Neurodegenerative, Psychiatric and Proliferative Disorders. Int J Mol Sci. 2013;14: 10122–10142. doi:10.3390/ijms140510122

17. Hirata Y, Kobayashi T, Nishiumi S, Yamanaka K, Nakagawa T, Fujigaki S, et al. Identification of highly sensitive biomarkers that can aid the early detection of pancreatic cancer using GC/MS/MS-based targeted metabolomics. Clin Chim Acta. 2017;468: 98–104. doi:10.1016/j.cca.2017.02.011

18. Ueda HH, Nagasawa Y, Sato A, Onda M, Murakoshi H. Chronic neuronal excitation leads to dual metaplasticity in the signaling for structural long-term potentiation. Cell Rep. 2022;38: 110153. doi:10.1016/j.celrep.2021.110153

19. Qiao S, Iwashita T, Furukawa T, Yamamoto M, Sobue G, Takahashi M. Differential Effects of Leukocyte Common Antigen-related Protein on Biochemical and Biological Activities of RET-MEN2A and RET-MEN2B Mutant Proteins*. J Biol Chem. 2001;276: 9460–9467. doi:10.1074/jbc.M008744200

20. Zhu S, Zhao C, Wu Y, Yang Q, Shao A, Wang T, et al. Identification of a Vav2-dependent mechanism for GDNF/Ret control of mesolimbic DAT trafficking. Nat Neurosci. 2015;18: 1084–1093. doi:10.1038/nn.4060

21. Mandt BH, Zahniser NR. Low and high cocaine locomotor responding male Sprague-Dawley rats differ in rapid cocaine-induced regulation of striatal dopamine transporter function. Neuropharmacology. 2010;58: 605. doi:10.1016/j.neuropharm.2009.11.015

22. Bernstein DL, Lewandowski SI, Besada C, Place D, España RA, Mortensen OV. Inactivation of ERK1/2 Signaling in Dopaminergic Neurons by Map Kinase Phosphatase MKP3 Regulates Dopamine Signaling and Motivation for Cocaine. J Neurosci. 2024;44. doi:10.1523/JNEUROSCI.0727-23.2023

23. Cortés BI, Meza RC, Ancatén-González C, Ardiles NM, Aránguiz M-I, Tomita H, et al. Loss of protein tyrosine phosphatase receptor delta PTPRD increases the number of cortical neurons, impairs synaptic function and induces autistic-like behaviors in adult mice. Biol Res. 2024;57: 40. doi:10.1186/s40659-024-00522-0

24. Lanuza MA, Just-Borràs L, Hurtado E, Cilleros-Mañé V, Tomàs M, Garcia N, et al. The Impact of Kinases in Amyotrophic Lateral Sclerosis at the Neuromuscular Synapse: Insights into BDNF/TrkB and PKC Signaling. Cells. 2019;8: 1578. doi:10.3390/cells8121578

25. Gabriel LR, Wu S, Kearney P, Bellvé KD, Standley C, Fogarty KE, et al. Dopamine Transporter Endocytic Trafficking in Striatal Dopaminergic Neurons: Differential Dependence on Dynamin and the Actin Cytoskeleton. J Neurosci. 2013;33: 17836–17846. doi:10.1523/JNEUROSCI.3284-13.2013

26. Daniels GM, Amara SG. Regulated Trafficking of the Human Dopamine Transporter. J Biol Chem. 1999;274: 35794–35801. doi:10.1074/jbc.274.50.35794

27. Sorkina T, Hoover BR, Zahniser NR, Sorkin A. Constitutive and Protein Kinase C-Induced Internalization of the Dopamine Transporter is Mediated by a Clathrin-Dependent Mechanism. Traffic. 2005;6: 157–170. doi:10.1111/j.1600-0854.2005.00259.x

28. Chen R, Furman CA, Zhang M, Kim MN, Gereau RW, Leitges M, et al. Protein Kinase Cβ Is a Critical Regulator of Dopamine Transporter Trafficking and Regulates the Behavioral Response to Amphetamine in Mice. J Pharmacol Exp Ther. 2009;328: 912–920. doi:10.1124/jpet.108.147959

29. Johnson LA, Furman CA, Zhang M, Guptaroy B, Gnegy ME. Rapid delivery of the dopamine transporter to the plasmalemmal membrane upon amphetamine stimulation. Neuropharmacology. 2005;49: 750–758. doi:10.1016/j.neuropharm.2005.08.018

30. Liu Y, Sarkar A, Kheradpour P, Ernst J, Kellis M. Evidence of reduced recombination rate in human regulatory domains. Genome Biol. 2017;18: 193. doi:10.1186/s13059-017-1308-x

31. Werme J, van der Sluis S, Posthuma D, de Leeuw CA. An integrated framework for local genetic correlation analysis. Nat Genet. 2022;54: 274–282. doi:10.1038/s41588-022-01017-y
